# Bace1-dependent amyloid processing regulates hypothalamic leptin sensitivity in obese mice

**DOI:** 10.1038/s41598-017-18388-6

**Published:** 2018-01-08

**Authors:** Paul J. Meakin, Susan M. Jalicy, Gemma Montagut, David J. P. Allsop, Daniella L. Cavellini, Stuart W. Irvine, Christopher McGinley, Mary K. Liddell, Alison D. McNeilly, Karolina Parmionova, Yu-Ru Liu, Charlotte L. S. Bailey, J. Kim Dale, Lora K. Heisler, Rory J. McCrimmon, Michael L. J. Ashford

**Affiliations:** 1 Division of Molecular and Clinical Medicine, School of Medicine, Ninewells Hospital & Medical School, Dundee, DD1 9SY UK; 2 School of Life Sciences, University of Dundee, Dundee, DD1 5EH UK; 3 Rowett Institute of Nutrition and Health, Aberdeen, AB21 9SB UK

## Abstract

Obesity places an enormous medical and economic burden on society. The principal driver appears to be central leptin resistance with hyperleptinemia. Accordingly, a compound that reverses or prevents leptin resistance should promote weight normalisation and improve glucose homeostasis. The protease Bace1 drives beta amyloid (Aβ) production with obesity elevating hypothalamic Bace1 activity and Aβ_1–42_ production. Pharmacological inhibition of Bace1 reduces body weight, improves glucose homeostasis and lowers plasma leptin in diet-induced obese (DIO) mice. These actions are not apparent in *ob/ob* or *db/db* mice, indicating the requirement for functional leptin signalling. Decreasing Bace1 activity normalises hypothalamic inflammation, lowers PTP1B and SOCS3 and restores hypothalamic leptin sensitivity and pSTAT3 response in obese mice, but does not affect leptin sensitivity in lean mice. Raising central Aβ_1–42_ levels in the early stage of DIO increases hypothalamic basal pSTAT3 and reduces the amplitude of the leptin pSTAT3 signal without increased inflammation. Thus, elevated Aβ_1–42_ promotes hypothalamic leptin resistance, which is associated with diminished whole-body sensitivity to exogenous leptin and exacerbated body weight gain in high fat fed mice. These results indicate that Bace1 inhibitors, currently in clinical trials for Alzheimer’s disease, may be useful agents for the treatment of obesity and associated diabetes.

## Introduction

Long-term disordered glucose and lipid metabolism are significant risk factors for cardiovascular disease and dementia and are major obstacles to sustainable health in an aging population. Consequently, understanding the pathways and mechanisms that underpin dietary-induced metabolic disorder is critically important. The aspartyl protease, Bace1 (beta-site amyloid precursor protein (APP)-cleaving enzyme 1) catalyzes the rate-limiting step in Aβ production^1^. Human Aβ peptides are prone to aggregation causing amyloid plaques, a process strongly linked^2^ to Alzheimer’s disease (AD) and elevated brain Bace1 correlates with amyloid pathology in AD mouse models^3^. People with late-onset (sporadic) AD exhibit central insulin resistance and glucose intolerance with an increased risk of type 2 diabetes (T2D)^4^. Conversely peripheral insulin resistance strongly correlates with higher risk of AD^5^. Thus, the pathological processes underlying AD may be linked with the metabolic disturbances associated with T2D and obesity. Indeed, mice genetically deficient in Bace1^6^ exhibit improved glucose homeostasis and insulin sensitivity and are resistant to diet-induced obesity (DIO), whereas mice with a neuronal-specific knock-in of human Bace1 display a systemic diabetic phenotype^7^.

Circulating leptin is an important signal of stored energy availability, with fasting-driven reduced plasma leptin sufficient to engage compensatory mechanisms to restore feeding and maintain body mass. To suppress appetite and increase energy expenditure, leptin modifies hypothalamic function by altering transcription of arcuate nucleus (ARC) orexigenic (neuropeptide Y (NPY) and agouti-related protein (AgRP)) and anorexigenic (proopiomelanocortin (POMC) and cocaine- and amphetamine-regulated transcript (CART)) peptides and neuronal excitability^8,9^. Chronic high fat feeding, leading to obesity, increases leptin expression in proportion to adipose tissue mass^10^ resulting in high plasma leptin. In obesity, hypothalamic leptin sensitivity is reduced causing failure to adequately suppress food intake and increase energy expenditure (i.e. leptin sensing circuitry is insensitive to a pharmacological dose of leptin; termed leptin resistance). The initial and predominating site for high fat diet (HFD)-induced leptin resistance is the ARC, with other nuclei (e.g. ventromedial (VMH), dorsomedial (DMH) and paraventricular (PVH) hypothalamus) demonstrating progressive and selective loss of leptin sensitivity^11–13^. The molecular and cellular mechanisms underlying induction of hypothalamic leptin resistance are unclear, although inflammation^14–16^ and endoplasmic reticulum (ER) stress^14,17,18^ are thought to activate signalling cascades that limit hypothalamic leptin sensitivity.

At the cellular level, hypothalamic leptin signalling is assessed through measurement of phosphorylated STAT3^Tyr705^ (pSTAT3^Tyr705^) levels, with DIO mediated leptin resistance associated with reduced response amplitude^19^. Impairment of STAT3 signalling through the long form of the leptin receptor (LepR-b) is associated with up-regulation of negative regulators of leptin signalling^20^, notably suppressor of cytokine signalling 3 (SOCS3) and protein tyrosine phosphatase 1B (PTP1B). Although leptin resistance in rodents and humans is reversed by dietary modification, inducements to reduce calorie intake and increase exercise have not proved particularly successful in most populations where obesity levels are high. Thus, finding a way to block or reverse the processes that mediate hypothalamic leptin signal attenuation is an attractive therapeutic strategy to reduce obesity and improve metabolic homeostasis.

This study was designed to examine the premise that consumption of chronic high fat diet, sufficient to cause obesity in mice, increases neuronal Bace1 activity and Aβ peptide levels, resulting in modification of the processes that act to limit hypothalamic leptin sensitivity resulting in body weight gain and loss of glucose homeostasis.

## Results

### Bace1 is expressed in the hypothalamus

**Figure 1:**
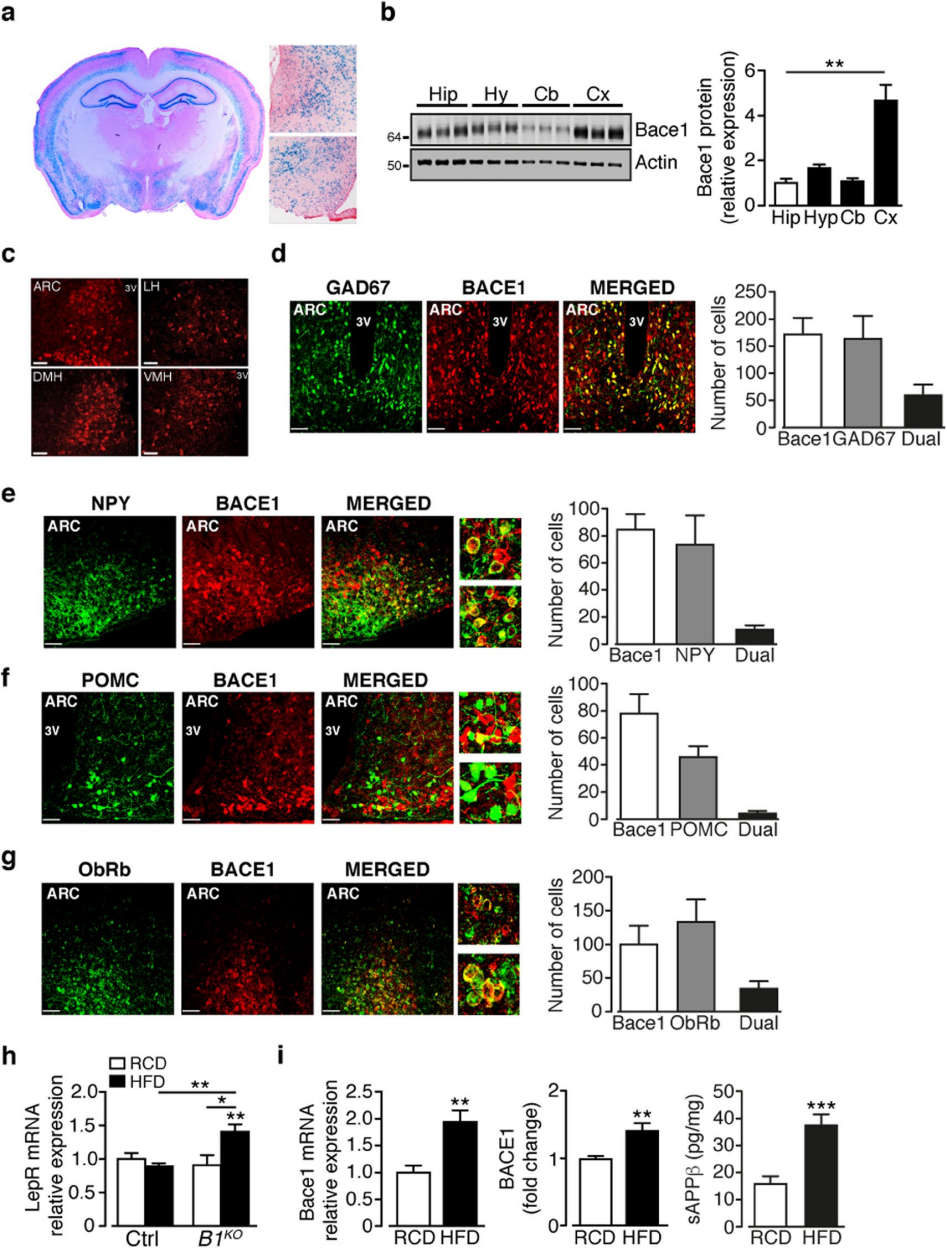
Bace1 is present in hypothalamic neurons and HFD increases Bace1 levels and activity. (**a**) Coronal section from *Bace1* ^*KO*^ brain depicting α-galactosidase staining. Higher magnification images show staining in DMH (upper) and ARC/VMH (lower). (**b**) Bace1 protein levels in brain regions of control mice with signal intensity quantified against actin and normalised to hippocampus (n = 15/group). (**c**) Representative images of Bace1 immunoreactivity in ARC, LH, DMH and VMH. Bars, 50 µm. (**d**–**g**) ARC Bace1 immunoreactivity in GAD67-GFP (**d**) n = 3 mice), NPY-GFP (**e**) n = 6 mice) and POMC-GFP (**f**) n = 5 mice), and LEPR-GFP (**g**) n = 4 mice) with merged images. Bars, 50 µm. Higher magnification images show close apposition between NPY (**e**), POMC (**f**) and Bace1 expressing neurons. Bar graphs for (**d**–**g**) show number of cells singly- and dual-labelled. (**h**) BMH LepR expression in RCD and HFD control and *Bace1* ^*KO*^ mice (n = 7–11). (**i**) BMH Bace1 mRNA and protein (n = 6–8) expression and activity (sAPPβ; n = 12–13) in RCD and HFD control mice. Data are means ± SEM. *P < 0.05, **P < 0.01, ***P < 0.001 by one-way ANOVA with Bonferroni’s multiple comparisons test (**b**,**h**) or 2-tailed unpaired Student’s t-test with Welch correction (**i**). Uncropped images of immunoblots can be found in Supplementary Fig. 9.

We examined brain Bace1 gene expression by β-galactosidase staining of coronal slices from Bace1 null (*Bace1* ^*KO*^) mice, which uses the endogenous murine Bace1 locus to drive LacZ^21^. Bace1 is widely expressed in the hippocampus and cortical lamina^21^ and in the hypothalamus, notably ARC, VMH and DMH (Fig. 1a). Bace1 protein was detected in mouse hypothalamus, with levels like hippocampus and cerebellum, but lower than cortex (Fig. 1b). Examination of Bace1 protein distribution demonstrates its presence in hypothalamic centres, with clusters of Bace1 positive cells in the ARC, lateral hypothalamus (LH), DMH and VMH (Fig. 1c). Bace1 was not detected in the hypothalamus (not shown) or other brain regions^6^ of *Bace1* ^*KO*^ mice.

Bace1 is localised to neurons (Supplementary Fig. 1a,b), with many ARC Bace1-containing neurons exhibiting co-localisation with GAD67 (~34%, Fig. 1d) or VGlut2 (~31%, Supplementary Fig. 1c). Dual-staining for Bace1 and GFP in NPY-hrGFP and POMC-eGFP mouse lines or staining for Bace1 with fluorescence *in situ* hybridisation for *Npy* and *Pomc*, revealed that few NPY (~12%) or POMC (~5%) neurons co-localise with Bace1 (Fig. 1e,f Supplementary Fig. 1d). However, many Bace1 containing neurons exhibit close apposition to POMC/CART and NPY/AgRP cell bodies or proximity to their nerve fibres (Fig. 1e,f expanded images). Thus, most ARC Bace1 positive neurons are presently of unknown neuropeptide phenotype. To determine whether leptin may have a direct effect on Bace1 containing neurons, we performed dual-labelling for Bace1 and LepR-b and found that ~35% of ARC Bace1 neurons express LepR-b (Fig. 1g). Similar results were obtained using the LEPR-GFP mouse line (Supplementary Fig. 1e). HFD raised *Lepr* expression in *Bace1* ^*KO*^ basomedial hypothalamus (BMH), but not in controls (Fig. 1h). In DIO mice BMH Bace1 mRNA, protein expression and activity are increased (Fig. 1i), the latter determined by the amount of the APP cleavage product^1^, soluble APPβ (sAPPβ).

### Food intake and leptin sensitivity in mice with genetic reduction of Bace1

**Figure 2:**
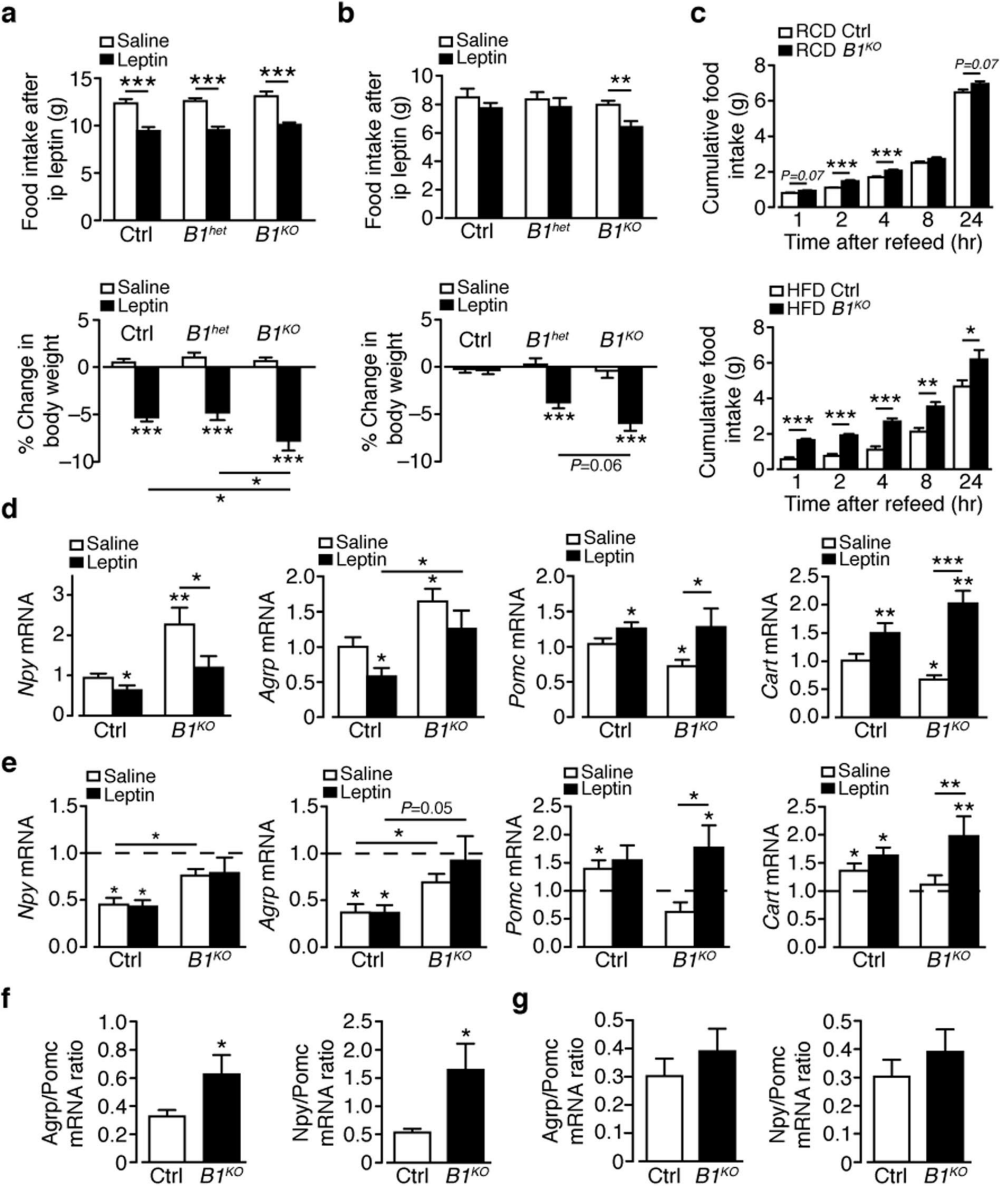
Bace1 alters leptin sensitivity and neuropeptide expression response to diet and leptin. Effects of 3-day vehicle or leptin (n = 7–14/group) on food intake and body weight change in control, *Bace1* ^*het*^ and *Bace1* ^*KO*^ mice on RCD (**a**) and HFD (**b**). (**c**) Cumulative 24-hour food intake in response to overnight fast in RCD (upper; n = 6–11 /group) and HFD (lower; n = 4–8/group) mice. (**d**,**e**) RT-qPCR of BMH Npy, Agrp, Pomc and Cart from saline or leptin-treated RCD (**d**) and HFD (**e**) control and *Bace1* ^*KO*^ mice (n = 6–11 mice). Data normalized to RCD controls (dashed line in **e**). Agrp/Pomc and Npy/Pomc ratios for RCD (**f**) and HFD (**g**) mice. Data are means ± SEM. *P < 0.05, **P < 0.01, P < 0.001 by one-way ANOVA with Bonferroni’s multiple comparisons test (**a**–**c**), Kruskal-Wallis test (**d**,**e**) or 2-tailed unpaired Student’s t-test (**f**,**g**).

Mice with no (*Bace1* ^*KO*^), or reduced (heterozygote; *Bace1* ^*het*^) Bace1 resist HFD mediated obesity^6^. To investigate whether this lean phenotype results from enhanced leptin sensitivity we assessed the effect of leptin (2 mg/kg, i.p.) on food intake and body weight in regular chow diet (RCD) *Bace1* ^*KO*^, *Bace1* ^*het*^ and control mice. Leptin suppressed food intake equally in all three genotypes, although *Bace1* ^*KO*^ mice displayed a greater reduction in body weight than *Bace1* ^*het*^ or control mice (Fig. 2a). This higher leptin sensitivity was unlikely determined by the leanness of *Bace1* ^*KO*^ mice, as they were weight-matched to controls. DIO mice exhibited no change in food intake or body weight in response to leptin (Fig. 2b), indicative of leptin resistance. In contrast, leptin reduced body weight but not food intake in HFD *Bace1* ^*het*^ mice (weight-matched to DIO control), with food intake and body weight decreased by leptin in HFD *Bace1* ^*KO*^ mice (Fig. 2b). Thus, reducing Bace1 levels improves leptin efficacy independent of the degree of adiposity. Cumulative food intake is unchanged between genotypes on both diets^6^, so as a more sensitive indicator we measured compensatory feeding after an overnight fast. *Bace1* ^*KO*^ mice displayed hyperphagia when re-fed, an effect exacerbated by HFD (Fig. 2c) suggesting a role for Bace1 in the response to fasting.

The melanocortin system, in part through the POMC product α-MSH and the melanocortin 4 receptor (MC4R), is strongly implicated in energy and glucose homeostasis^22^. To investigate whether this pathway, downstream of ARC neurons, is modified by Bace1 activity mice were treated with the non-selective MCR agonist, melanotan II (MT-II). After 20 weeks on RCD or HFD, *Bace1* ^*KO*^ mice displayed the same reduction in food intake in response to MT-II (50 µg, i.p.) as control mice (Supplementary Fig. 2a–c), indicating that lack of Bace1 does not alter the effectiveness of this agonist on second order hypothalamic neurons.

### Bace1 alters the effects of diet and leptin on hypothalamic neuropeptide expression

To understand the mechanisms through which Bace1 reduction lowers body weight and resists HFD mediated obesity, we first measured hypothalamic neuropeptide gene expression. On RCD and following overnight fast, BMH *Npy* and *Agrp* were elevated, and *Pomc* and *Cart* decreased (Fig. 2d) in *Bace1* ^*KO*^, compared to control mice. In RCD control mice, the expected reduction in *Npy* and *Agrp* and increase in *Pomc* and *Cart* (Fig. 2d) following leptin (3 mg/kg, i.p.) treatment were obtained^23^. *Bace1* ^*KO*^ mice displayed broadly similar responses to leptin with decreased *Npy* and increased *Pomc* and *Cart*, although *Agrp* was unaffected. In DIO mice, *Npy* and *Agrp* were suppressed and *Pomc* and *Cart* increased by the dietary challenge, with the (3 mg/kg, i.p.) leptin response blunted (Fig. 2e). *Bace1* ^*KO*^ mice displayed a reduced response to HFD retaining higher *Npy* and *Agrp*, lower *Pomc* and unaltered *Cart* compared to DIO mice, and leptin did not suppress *Npy* and *Agrp*, but increased *Pomc* and *Cart* (Fig. 2e). Thus, the orexigenic (*Npy*, *Agrp*) to anorexigenic (*Pomc*) ratio in the fasted state was increased in RCD *Bace1* ^*KO*^ mice (Fig. 2f), in line with hyperphagia observed in fast-refeeding, and suppressed by HFD (Fig. 2g), suggesting that Bace1 limits orexigenic drive induced by fasting in a diet dependent manner.

We next examined whether Bace1 altered neuropeptide expression or leptin sensitivity in hypothalamic regions important for energy homeostasis containing second order neurons, which are innervated by ARC neurons. Basal levels of *Crh*, *Trh, Pmch* and *Pro-Orexin* were unaltered in fasted *Bace1* ^*KO*^, compared with control mice, with leptin responsiveness unchanged except for *Pmch*, which was elevated in *Bace1* ^*KO*^ mice (Supplementary Fig. 2d). Lack of Bace1 did not alter fasted *Mc4r* or its increase by leptin (Supplementary Fig. 2d). Collectively, these data indicate that reducing Bace1 activity primarily alters the responsiveness of first order ARC neurons to dietary signals and their sensitivity to leptin.

### Hypothalamic leptin signalling is preserved in HFD *Bace1*^*KO*^ mice

**Figure 3:**
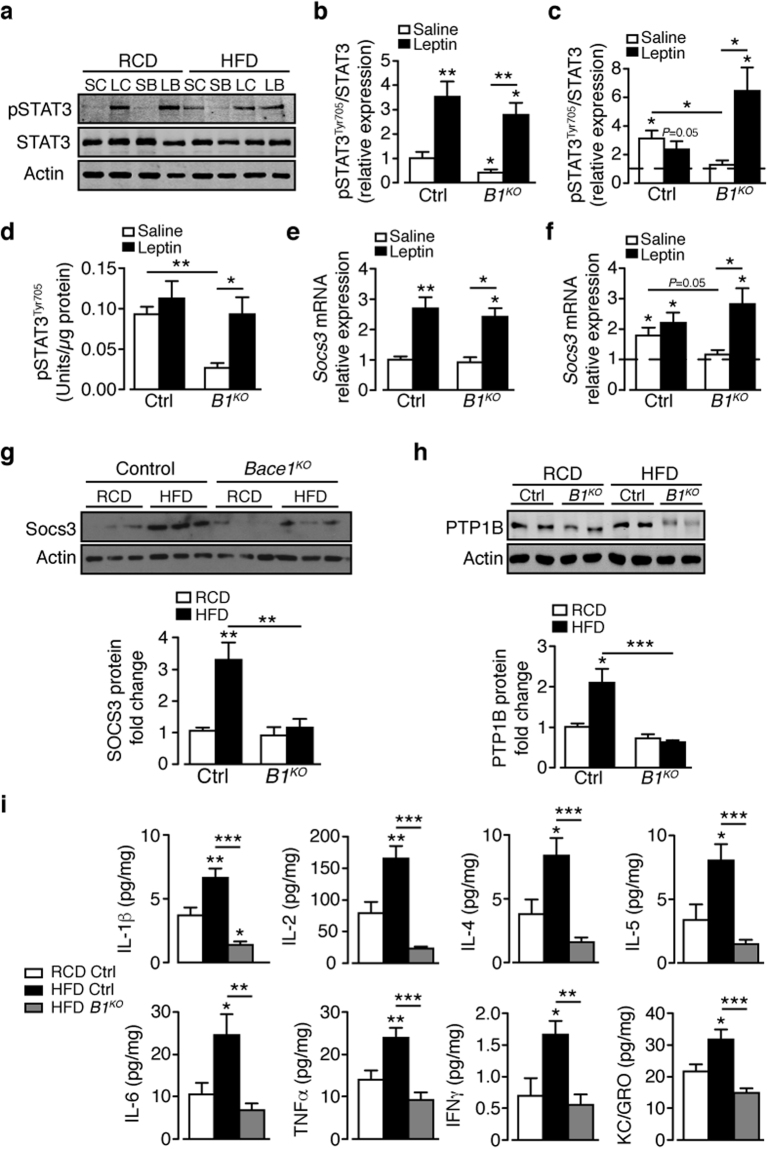
Absence of Bace1 enhances leptin signalling in DIO mice and prevents HFD-induced hypothalamic inflammation. (**a**) Immunoblots of total STAT3, actin and pSTAT3^Tyr705^ levels in saline- and leptin-stimulated control (SC, LC) and *Bace1* ^*KO*^ (SB, LB) mice on RCD or HFD. Ratio of signal intensities for pSTAT3^Tyr705^ to STAT3 in RCD (**b**) and HFD (**c**) mice (n = 6–9/group). (**d**) pSTAT3 by ELISA for RCD and HFD control (n = 7) and *Bace1* ^*KO*^ (n = 8) mice. (**e**,**f**) RT-qPCR for Socs3 from saline and leptin-treated control and *Bace1* ^*KO*^ mice (n = 6–10/group) on RCD (**e**) and HFD (**f**). (**g**,**h**) Immunoblots of hypothalamic SOCS3 (**g**) and PTP1B (**h**) and actin of control and *Bace1* ^*KO*^ mice on RCD and HFD. Quantified signal intensity of SOCS3 (n = 3–8/group) and PTP1B (n = 6–13/group) to actin. (**i**) Hypothalamic cytokine levels for RCD and HFD control and HFD *Bace1* ^*KO*^ mice (n = 11–14/group). Data are means ± SEM. *P < 0.05, **P < 0.01, P < 0.001 by Kruskal-Wallis test (**b**,**c**,**e**,**f**,**h**) or one-way ANOVA with Bonferroni’s multiple comparisons test (**d**,**i**). Uncropped images of immunoblots can be found in Supplementary Fig. 10.

To assess further how reduced Bace1 levels affect hypothalamic leptin signalling, we measured BMH pSTAT3^Tyr705^. For RCD, basal pSTAT3^Tyr705^ was lower in *Bace1* ^*KO*^ mice than controls, with the maximal pSTAT3^Tyr705^ level elicited by leptin unaltered (Fig. 3a,b). DIO mice exhibit raised basal p-STAT3^Tyr705^ with loss of response to leptin, as expected^11^ whereas HFD *Bace1* ^*KO*^ mice retain lower basal pSTAT3^Tyr705^ and remain sensitive to leptin (Fig. 3a,c). Identical results for vehicle and leptin-stimulated hypothalamic pSTAT3^Tyr705^ levels in HFD mice were obtained using a STAT3(pY^705^) ELISA (Fig. 3d).

Therefore, chronic HFD raises basal pSTAT3^Tyr705^ and decreases leptin responsiveness in control mice, effects averted in the absence of Bace1 and correlated with reduced adiposity and lower plasma leptin^6^. Basal *Socs3* was unaltered in RCD control and *Bace1* ^*KO*^ mice and equally elevated following leptin treatment (3 mg/kg, i.p.; Fig. 3e), as expected from the pSTAT3^Tyr705^ increment. HFD raised *Socs3* in control mice with loss of stimulation by leptin, whereas HFD *Bace1* ^*KO*^ mice exhibited basal *Socs3* levels, which were increased by leptin (Fig. 3f), mirroring the changes of pSTAT3^Tyr705^. Similarly, HFD increased hypothalamic SOCS3 levels in control but not *Bace1* ^*KO*^ mice (Fig. 3g). Hypothalamic PTP1B levels were raised in DIO mice, whereas in *Bace1* ^*KO*^ mice PTP1B was lowered and insensitive to HFD (Fig. 3h).

Recent studies indicate that HFD increases hypothalamic inflammation and this may drive leptin resistance^24^. ER stress signalling responses also contribute to adaptation pathways linking nutrient metabolism and inflammation^25^, and inhibition of hypothalamic ER stress restores leptin sensitivity and decreases food intake^17^. Raised levels of inflammatory cytokines were detected in the hypothalamus of HFD versus RCD control mice, but not in HFD *Bace1* ^*KO*^ mice (Fig. 3i). After 20 weeks HFD, although some ER stress response transcript levels were reduced in *Bace1* ^*KO*^ hypothalamus, no marked changes in protein levels were seen (Supplementary Fig. 3a,b) indicating there was no consistent activation of ER stress pathways. These data suggest that, under conditions of chronic HFD, Bace1 absence prevents up-regulation of hypothalamic inflammatory cytokine levels independent of ER stress and maintains lowered PTP1B and SOCS3, all of which may contribute to the preserved leptin signalling and lean phenotype of the HFD *Bace1* ^*KO*^ mouse.

### Bace1 inhibition requires intact leptin signalling to reduce body weight and improve glucose homeostasis in obese mice

**Figure 4:**
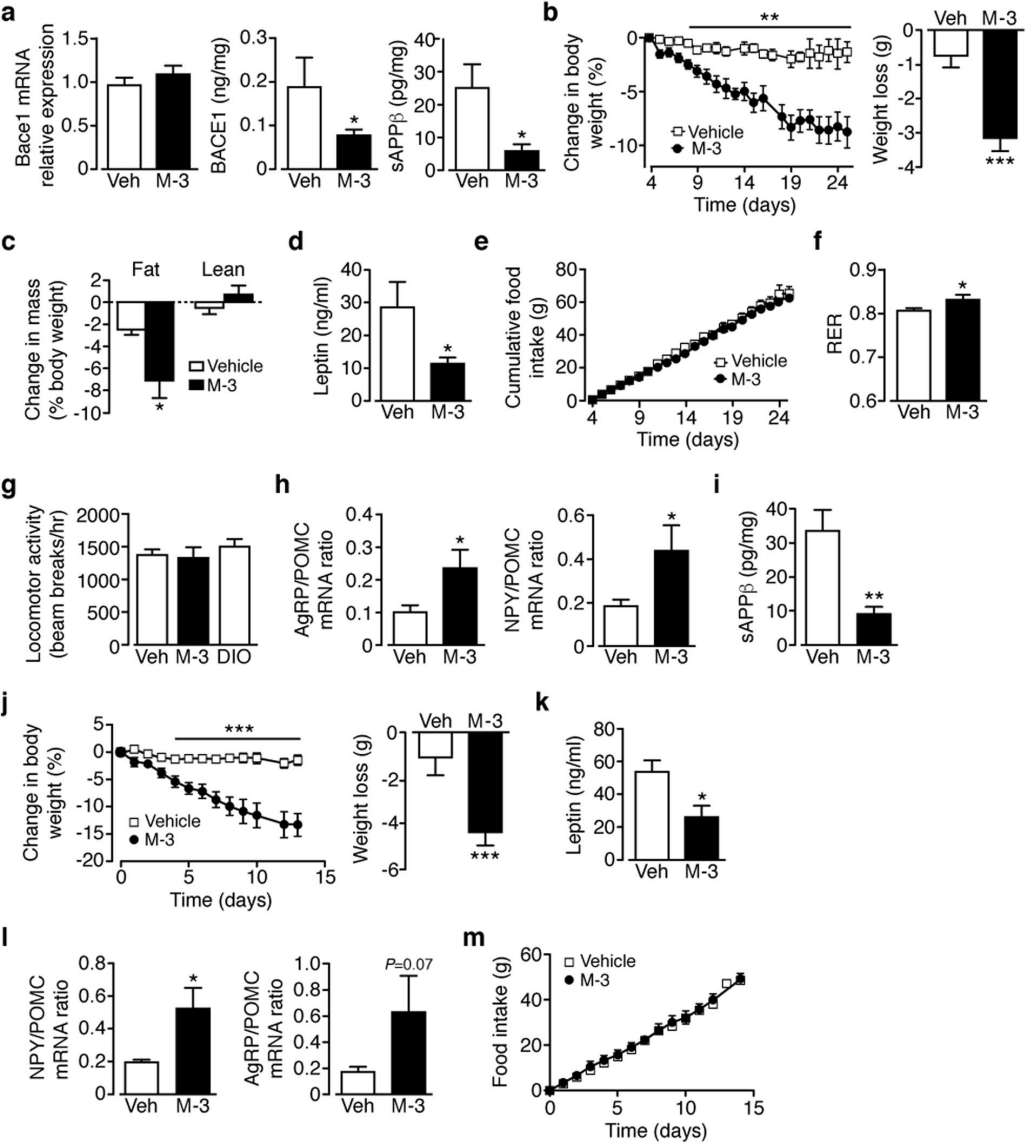
Peripheral or central administration of Bace1 inhibitor reduces body weight and hyperleptinemia of DIO mice. (**a**) Hypothalamic Bace1 mRNA (n = 9–11), protein (n = 7–11) and activity (sAPPβ; n = 8) in sc vehicle or M-3 DIO mice. (**b**) Percentage body weight decrease and actual weight loss in DIO mice given sc vehicle or M-3 (n = 11). (**c**) qMR of DIO mice after sc vehicle or M-3 (n = 6–7/group). (**d**) Plasma leptin of DIO mice after sc vehicle or M-3 (n = 8–9/group). (**e**) Cumulative food intake of DIO mice given sc vehicle or M-3 (n = 11). (**f**) RER of DIO mice after sc vehicle or M-3 (n = 5–7/group). (**g**) Locomotor activity of naïve DIO mice and DIO mice after sc vehicle or M-3 (n = 6–7/group). (**h**) Agrp/Pomc and Npy/Pomc ratios for DIO mice after sc vehicle or M-3 (n = 12–14/group). (**i**) Hypothalamic sAPPβ of icv vehicle or M-3 DIO mice (n = 11–12/group). (**j**) Percentage body weight decrease and actual weight loss in DIO mice given icv vehicle or M-3 (n = 11). (**k**) Plasma leptin of DIO mice after icv vehicle or M-3 (n = 4–6/group). (**l**) Agrp/Pomc and Npy/Pomc ratios for DIO mice following icv vehicle or M-3 (n = 12–13/group). (**m**) Cumulative food intake of DIO mice given icv vehicle or M-3 (n = 7–9/group). Data are means ± SEM. *P < 0.05, **P < 0.01, ***P < 0.001 by 2-tailed unpaired Student’s t-test (**a**,**d**,**f**,**h**,**i**,**k**,**l**) body weight loss in (**b**,**j**), one-way ANOVA with Bonferroni’s multiple comparisons test (**c**,**g**) or repeated measures ANOVA with Sidak’s multiple comparisons test (**b**,**e**,**j**,**m**).

Next, we determined whether pharmacological inhibition of Bace1 reduces body weight and improves glucose homeostasis of DIO mice, emulating genetic reduction^6^. DIO mice were treated with the Bace1 inhibitor M-3^6^ or vehicle delivered subcutaneously (sc; 10 mg/kg/day) by osmotic minipump. The effectiveness of M-3 was verified by lowered levels of Bace1 protein (not mRNA) and sAPPβ (Fig. 4a). M-3 reduced body weight (Fig. 4b) and fat mass with no change in lean mass (Fig. 4c) and decreased plasma leptin (Fig. 4d) but did not alter food intake (Fig. 4e) of DIO mice. M-3 increased the respiratory exchange ratio (Fig. 4f), indicating raised carbohydrate oxidation, with no change in locomotor activity (Fig. 4g), as reported for *Bace1* ^*KO*^ mice^6^. An increased orexigenic to anorexigenic neuropeptide ratio in the fasted state (Fig. 4h) was detected in M-3 treated mice, indicative of an increased orexigenic drive. M-3 improved glucose tolerance, lowered fasted glucose and enhanced insulin sensitivity with a trend for lower insulin in DIO mice (Supplementary Fig. 4a–d). As peripheral M-3 (200 pmol/ml in serum) reduced hypothalamic Bace1 activity and was detectable in whole brain homogenates (250 pmol/g wet weight) we determined whether intracerebroventricular (icv) delivery would replicate these metabolic improvements. M-3 given icv to DIO mice for 14 days reduced hypothalamic Bace1 activity (Fig. 4i), decreased body weight (Fig. 4j), lowered plasma leptin (Fig. 4k) and increased neuropeptide ratio in the fasted state (Fig. 4l) with no change in food intake (Fig. 4m). M-3 icv improved glucose disposal (Supplementary Fig. 4e), but did not affect plasma insulin or peripheral insulin sensitivity (Supplementary Fig. 4f,g). Consequently, central M-3 replicates most of the metabolic outcomes observed for peripheral M-3 administration.

Although M-3 treatment of DIO mice phenocopied the effects observed in HFD *Bace1* ^*KO*^ mice, we repeated the experiment using a structurally different Bace1 inhibitor, AZ-4217^26^. DIO mice were treated orally (50 mg/kg/day) for 28 days with AZ-4217 or vehicle. AZ-4217 lowered hypothalamic Bace1 activity, did not affect food intake, reduced body weight and fat mass with no change in lean mass (Supplementary Fig. 5a–d) and improved glucose disposal and reduced fasting blood glucose (Supplementary Fig. 5e,f). AZ-4217 diminished the hyperleptinemia but not the hyperinsulinemia in DIO mice (Supplementary Fig. 5g). In RCD control mice, M-3 (sc; 10 mg/kg/day) had no effect on food intake, body weight, insulin sensitivity, plasma leptin or insulin levels (Supplementary Fig. 6a–d), but improved glucose tolerance with raised fasted glucose (Supplementary Fig. 6e,f). To determine whether the effects of Bace1 inhibition are leptin-signalling dependent, we treated leptin-deficient *ob/ob* (Supplementary Fig. 7a–f) and leptin receptor defective *db/db* (Supplementary Fig. 7g–l) mice with M-3 (sc; 10 mg/kg/day). Although M-3 reduced hypothalamic Bace1 activity (Supplementary Fig. 7a,g), there was no change in food intake (not shown), body weight or fat mass (Supplementary Fig. 7b,h), glucose disposal (Supplementary Fig. 7c,i), plasma insulin levels or insulin sensitivity (Supplementary Fig. 7d,e,j). M-3 treatment raised fasted glucose in *ob/ob* but not *db/db* mice (Supplementary Fig. 7f,k) and reduced plasma leptin in *db/db* mice (Supplementary Fig. 7l). Taken together these results indicate that M-3 requires leptin and functional leptin receptor signalling to reduce body weight and improve glucose homeostasis, leading us to consider the possibility that Bace1 inhibition potentiates leptin action or re-sensitises obese (leptin resistant) mice to endogenous leptin.

### Inhibition of Bace1 activity reverses hypothalamic leptin resistance

**Figure 5:**
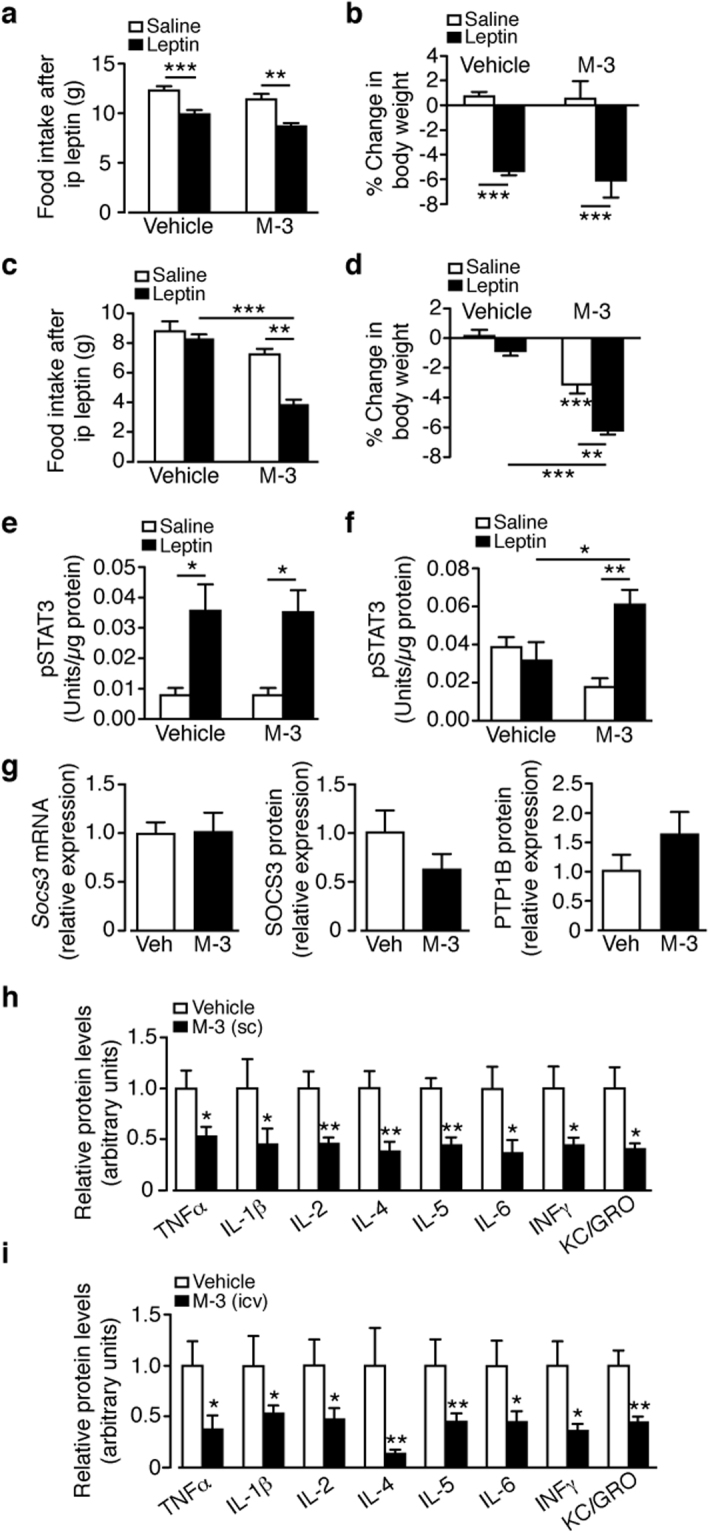
Bace1 Inhibition recovers leptin sensitivity in DIO mice and reduces inflammation. Vehicle or M-3 (10 mg/kg) administered to (**a**,**b**) lean (n = 5–18/group) or (**c**,**d**) DIO (n = 7–14/group) mice for 5 days, each group given saline or leptin (2 mg/kg) twice daily for the last 3 days. Food intake (**a**,**c**) and % body weight change (**b**,**d**) at end of injection period. Hypothalamic pSTAT3^Tyr705^ levels by ELISA for vehicle or M-3 treated mice receiving saline or leptin. (**e**) lean (n = 6–7/group) and (**f**) DIO (n = 7–8/group). (**g**) Hypothalamic levels of SOCS3, SOCS3 and PTP1B of DIO mice given vehicle or M-3 (n = 7–9, SOCS3 mRNA and protein; n = 5, PTP1B). (**h**,**i**) Relative cytokine levels of DIO hypothalamus after 14 days vehicle or sc M-3 (**h**) or icv M-3 (**i**) (n = 9–12/group). Data are means ± SEM. *P < 0.05, **P < 0.01, ***P < 0.001 by one-way ANOVA with Bonferroni’s multiple comparisons test (**a**–**f**) or 2-tailed unpaired Student’s t-test (**g**,**h**,**i**).

The inability of M-3 to reduce body weight of lean mice may be owing to the low levels of circulating leptin. Consequently, we examined whether lean mice pre-treated with M-3 elicited a stronger response to a pharmacological dose of leptin in comparison to lean mice pre-treated with vehicle. Mice were administered M-3 (sc; 10 mg/kg) or vehicle by daily i.p. injection for 5 days, the first 2 days with no leptin, then leptin (2 mg/kg) or saline twice daily for 3 days. Leptin administration to lean mice receiving vehicle decreased food intake and reduced body weight (Fig. 5a,b). Mice receiving M-3 with saline injection showed no change in food intake or body weight whereas the group given leptin and M-3 exhibited reduced food intake and decreased body weight, indistinguishable from vehicle + leptin controls (Fig. 5a,b). These results show that M-3 does not potentiate leptin action at normal circulating levels, or at a pharmacological dose of leptin, in lean mice.

To investigate whether M-3 re-sensitises obese mice to leptin, this experiment was repeated on DIO mice. Administration of leptin to DIO mice receiving vehicle did not alter food intake or body weight, as expected for leptin resistant mice (Fig. 5c,d). DIO mice receiving M-3 alone exhibited reduced body weight, but not food intake. Leptin given to DIO mice receiving M-3 caused a large reduction in food intake and body weight, greater than M-3 alone (Fig. 5c,d). These data suggest that Bace1 inhibition by M-3 re-sensitises obese mice to the effects of endogenous high levels of leptin, thus circumventing the process that causes leptin resistance.

To gain insight into mechanisms by which lowered Bace1 activity recovers leptin sensitivity in DIO mice, we examined hypothalamic pSTAT3^Tyr705^. Lean mice displayed no difference in basal or leptin stimulated levels of pSTAT3^Tyr705^ following vehicle or M-3 treatment (Fig. 5e). DIO mice that received vehicle demonstrated no change in pSTAT3^Tyr705^ levels in response to leptin whereas DIO mice pre-treated with M-3 exhibited increased levels of pSTAT3^Tyr705^ to leptin (Fig. 5f) indicating that acute treatment with M-3 is sufficient to salvage leptin sensitivity. M-3 treatment of DIO mice had no effect on *Socs3* or SOCS3 and PTP1B protein levels (Fig. 5g). There were no changes in hypothalamic ER stress markers in M-3 treated DIO mice (data not shown), although peripheral (Fig. 5h) or central (Fig. 5i) M-3 reduced hypothalamic inflammatory cytokine levels. Hence prevention or reversal of HFD-induced hypothalamic inflammation may be the mechanism by which reducing Bace1 activity restores leptin sensitivity^24^. Next, we sought to identify the molecular entity by which raised Bace1 activity reduces hypothalamic leptin sensitivity in DIO mice.

### Aβ_1–42_ exacerbates HFD-mediated weight gain by induction of hypothalamic leptin resistance

**Figure 6:**
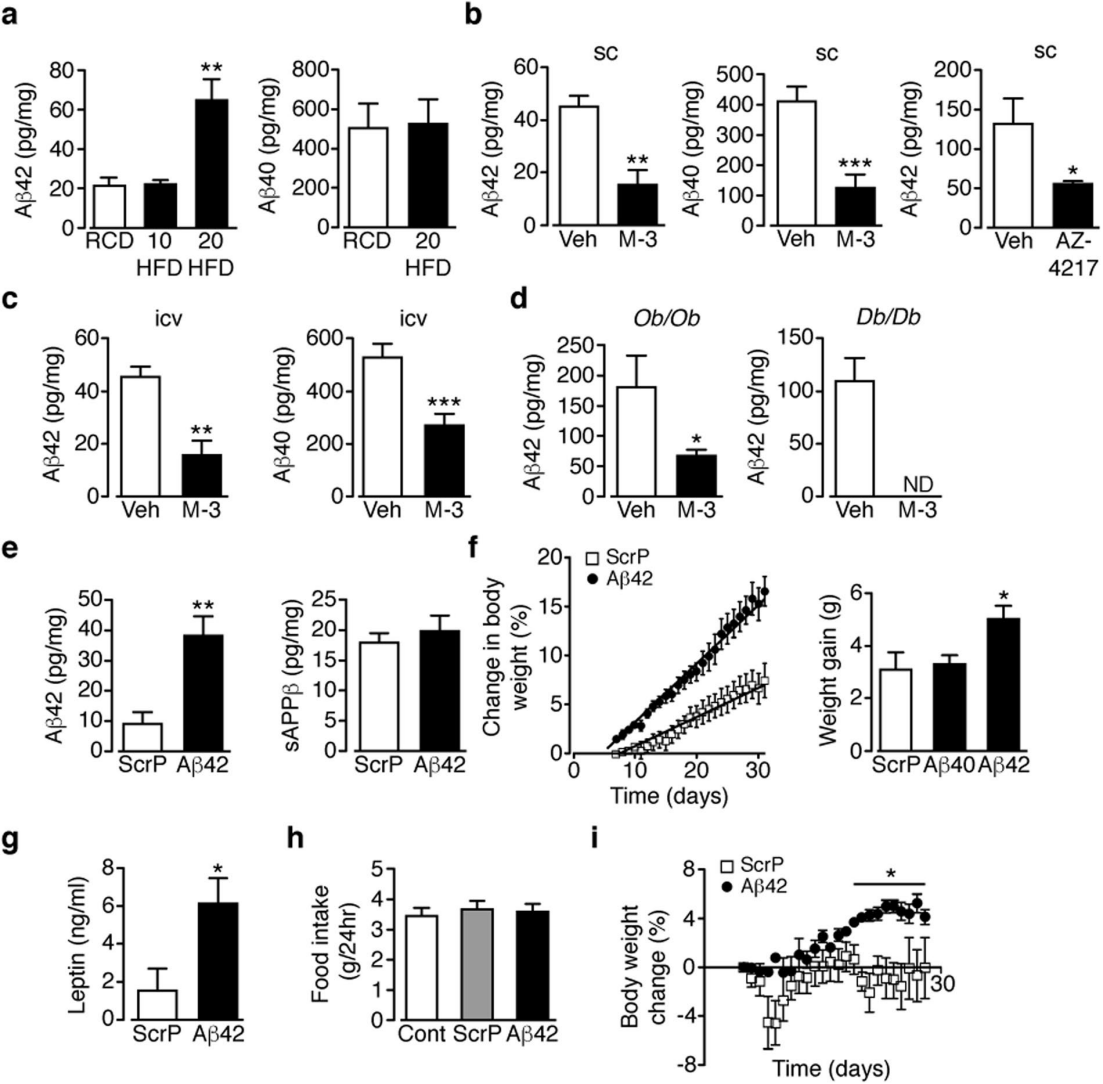
Central Aβ_1–42_ increases body weight and fat mass of HFD mice. (**a**) Hypothalamic Aβ_1–42_ after 20 weeks RCD (n = 9) and 10 (n = 7) and 20 (n = 10) weeks HFD, and Aβ_1–40_ after 20 weeks RCD (n = 11) and HFD (n = 11). (**b**,**c**) Hypothalamic Aβ_1–42_ and Aβ_1–40_ in DIO mice after sc vehicle, sc M-3 or oral AZ-4217 (**b**) and (**c**) icv M-3 for 14 days (n = 7–12/group). (**d**) Hypothalamic Aβ_1–42_ in *ob/ob* and *db/db* mice after sc vehicle or M-3 (n = 4–7). (**e**) Hypothalamic Aβ_1–42_ and sAPPβ after 10 weeks HFD then 28 days icv ScrP or Aβ_1–42_ (n = 5). (**f**) Percentage increase in body weight in HFD mice given icv ScrP or Aβ_1–42_ and actual weight increase for mice given icv ScrP, Aβ_1–42_ or Aβ_1–40_ (n = 9–17/group). (**g**) Plasma leptin after icv treatment with ScrP or Aβ_1–42_ (n = 10). (**h**) Food intake of HFD mice given ScrP or Aβ_1–42_, compared to non-treated controls (n = 13–15/group). (**i**) Percentage body weight change in RCD mice given icv ScrP or Aβ_1–42_ (n = 6). Data are means ± SEM. *P < 0.05, **P < 0.01, ***P < 0.001 by one-way ANOVA (**a**,**f**,**h**) or two-way ANOVA with Bonferroni’s multiple comparisons test (**f**,**i**) or 2-tailed unpaired Student’s t-test ((**b**–**e**,**g**) body weight gain in **f**).

The major substrate of Bace1 is APP; cleavage of which produces sAPPβ with further cleavage of the membrane stub by γ-secretase forming Aβ peptides, of which Aβ_1–40_ and Aβ_1–42_ are the main products^1^. Aβ_1–40_ and Aβ_1–42_ have been implicated as mediators or amplifiers of central ER stress and neuroinflammation^27,28^ and increased basal pSTAT3^29^. Hypothalamic Aβ_1–42_, but not Aβ_1–40_, is raised in mice fed HFD for 20 weeks, compared to age-matched RCD mice (Fig. 6a). Obese (DIO, *ob/ob* and *db/db*) mice given M-3 or AZ-4217 exhibit lowered hypothalamic Aβ_1–40_ and Aβ_1–42_ levels (Fig. 6b–d). The finding that hypothalamic Aβ_1–42_ and not Aβ_1–40_ is sensitive to HFD supports the hypothesis that Aβ_1–42_ may play an important role in modulating leptin signalling.

**Figure 7:**
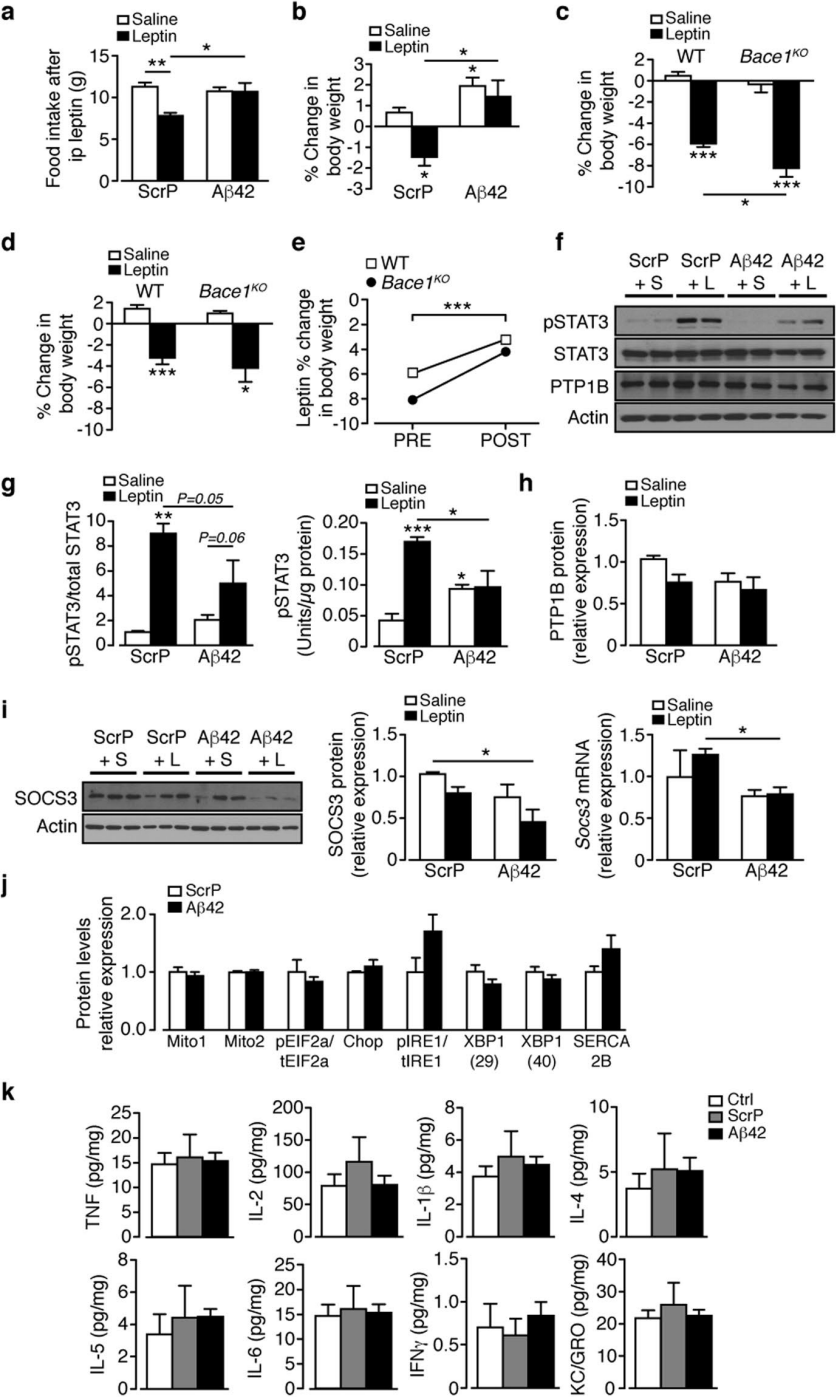
Central Aβ_1–42_ decreases hypothalamic leptin sensitivity of HFD mice. (**a**,**b**) ScrP or Aβ_1–42_ (3.36 µg/kg) given to HFD mice (starting at week 6) for 35 days, then given saline or leptin (2 mg/kg) twice daily for 3 days (n = 8–9/group). Food intake (**a**) and percentage body weight change (**b**) at end of injection period. (**c**) RCD WT (n = 7/group) and *Bace1* ^*KO*^ (n = 4/group) mice given saline or leptin (2 mg/kg) twice daily for 3 days and percentage body weight change determined. (**d**) 7 days later icv osmotic minipumps were inserted and Aβ_1–42_ (10 µg/kg) given to all mice for 21 days, then leptin sensitivity tested as above over the last 3 days. (**e**) leptin responsiveness of WT and *Bace1* ^*KO*^ mice before (PRE) and after (POST) Aβ_1–42_ treatment. (**f**) Immunoblots for saline and leptin-stimulated pSTAT3^Tyr705^, STAT3, PTP1B, and actin of HFD mice given ScrP or Aβ_1–42_, then injected with saline or leptin. (**g**) Ratio of signal intensities for pSTAT3^Tyr705^ to STAT3 (n = 5–7/group) and pSTAT3^Tyr705^ (n = 6–10/group) by ELISA in ScrP and Aβ_1–42_ treated mice after injection of saline or leptin. (**h**) Quantified signal intensity of PTP1B to actin for mice given ScrP or Aβ_1–42_ after injection of saline or leptin (n = 5–7/group). (**i**) Immunoblots of SOCS3 and actin of HFD mice given ScrP or Aβ_1–42_ after injection of saline or leptin, with quantified signal shown (n = 5–7/group). RT-qPCR for Socs3 for mice given ScrP and Aβ_1–42_ after injection of saline or leptin (n = 5–6/group), (**j**) Hypothalamic relative protein levels of ER stress markers in HFD mice given ScrP or Aβ_1–42_ (n = 5–7/group). (**k**) Hypothalamic cytokine levels by ELISA in HFD mice given ScrP or Aβ_1–42_ compared to untreated controls (n = 5–13/group). Data are means ± SEM. *P < 0.05, **P < 0.01, ***P < 0.001 by one-way ANOVA with Bonferroni’s multiple comparisons test (**a**,**b**,**c,d**,**g**,**k**), two-way ANOVA (e), Kruskal-Wallis test (**h**,**i**) or 2-tailed Student’s t-test with Welch correction (**j**). Uncropped images of immunoblots can be found in Supplementary Fig. 11.

Therefore, we tested the effects of icv mouse Aβ_1–42_ vs a scrambled peptide (ScrP) control (each @ 3.36 µg/kg) for 35 days on HFD mice, at a time (weeks 6–10) prior to hypothalamic Aβ_1–42_ levels being raised (Fig. 6a). The infusion of Aβ_1–42_ raised hypothalamic Aβ_1–42_ at 10 weeks to levels close to 20 weeks of HFD, with unaltered sAPPβ (Fig. 6e). The elevated Aβ_1–42_ exacerbated the response to HFD, whereby body weight (Fig. 6f) and plasma leptin (Fig. 6g) were increased in comparison to ScrP controls or as an additional control, Aβ_1–40_ -treated mice. The average daily food intake was unaltered by the form of the peptide infused (Fig. 6h), in comparison with 10 weeks HFD untreated mice. In RCD mice at an equivalent age, Aβ_1–42_ induced a much smaller increase in body weight (Fig. 6i), which was not associated with increased body fat or plasma leptin (not shown). Following the hypothesis that lowered Aβ_1–42_ is responsible for the recovery of leptin sensitivity in DIO mice, we reasoned that giving icv Aβ_1–42_ at this early time point, matching Aβ_1–42_ levels at 20 weeks of HFD, should promote hypothalamic leptin resistance. We infused Aβ_1–42_ or ScrP (3.36 µg/kg) icv to mice after 6 weeks of HFD and 35 days later, injected saline or leptin (2 mg/kg; 2x daily for 3 days) to each group. Leptin given to ScrP-infused mice decreased food intake (Fig. 7a) and body weight (Fig. 7b) compared to saline treated controls, indicative of partially preserved hypothalamic leptin sensitivity in the early stage of chronic HFD. In contrast, mice infused with Aβ_1–42_ and injected with saline showed no change in food intake and an increased body weight, with the Aβ_1–42_ group showing no response to leptin via food intake or body weight (Fig. 7a,b). These results suggest that the presence of Aβ_1–42_ resulted in an early (in relation to HFD time course) induction of leptin resistance. To examine the role of Bace1 in Aβ_1–42_ induced leptin resistance, we took RCD WT and *Bace1* ^*KO*^ mice and 7 days prior to icv minipump implantation determined their sensitivity (by change in body weight) to leptin (Fig. 7c), again demonstrating the higher sensitivity of *Bace1* ^*KO*^ mice to leptin. All mice were infused with Aβ_1–42_ for 21 days with an identical leptin sensitivity test performed over the last 3 days (Fig. 7d), resulting in diminished leptin responsiveness in both genotypes (Fig. 7e). This result supports the notion that elevated Bace1 activity regulates leptin signalling by cleavage of APP and increased production of Aβ_1–42_.

We next determined the effect of raised Aβ_1–42_ on pSTAT3^Tyr705^. HFD mice receiving ScrP and exogenous leptin exhibited increased hypothalamic pSTAT3^Tyr705^, compared to ScrP + saline treated mice, whereas HFD mice receiving Aβ_1–42_ demonstrated higher basal pSTAT3^Tyr705^ with no increase in response to leptin (Fig. 7f,g). This is consistent with the notion that raised Aβ_1–42_, notably in conjunction with consumption of a HFD, induces leptin resistance. We found no evidence of raised basal hypothalamic PTP1B or SOCS3 protein or mRNA levels associated with the Aβ_1–42_-induced leptin resistant state (Fig. 7f,h,i) and Aβ_1–42_ did not alter ER stress marker proteins in comparison to ScrP treated mice (Fig. 7j). Furthermore, no increase in hypothalamic inflammation (Fig. 7k) was detected at 10 weeks HFD and this was unaltered by the presence of ScrP or Aβ_1–42_ (values similar to 20 week RCD controls; see Fig. 3i). Collectively, these data indicate that raised hypothalamic Aβ_1–42_ at the early stages of HFD drives hypothalamic leptin resistance independently of up-regulation of negative regulators of leptin signalling or the induction of an inflammatory response.

## Discussion

Our results demonstrate that the consequence of lowered Bace1 activity in HFD mice is reduced body weight, fat mass and plasma leptin levels, which suggest an effect on hypothalamic leptin sensitivity. Bace1 is expressed in discrete groups of neurons in hypothalamic areas involved in energy homeostasis, with particularly high levels in the ARC. ARC Bace1 positive neurons are predominantly a mixture of GABA and glutamate containing neurons, but currently are poorly defined in terms of neuropeptide or alternative transmitter identity. However, it is likely that they contribute to leptin-sensitive ARC neuronal pathways and outputs as many Bace1 containing neurons are in close juxtaposition to NPY/AgRP and POMC/CART neurons and a significant proportion express LepR-b. Indeed, additional LepR-b expressing ARC populations, separate from POMC and NPY/AgRP, are known to contribute to leptin-sensitive circuit outputs^30^.

Loss of Bace1 had no impact on daily food intake on either RCD or HFD, thus the decreased body weight and fat mass in *Bace1* ^*KO*^ mice is likely accounted for by increased energy expenditure^6^. Nevertheless, augmented compensatory feeding was observed following fasting, an effect enhanced by HFD. DIO attenuates fasting induced hyperphagia by suppression of *Npy* and *Agrp* ^31^, an outcome also observed here. In contrast, lack of Bace1 altered the ARC neuropeptide transcription response to fasting in RCD mice, with higher *Npy* and *Agrp* and lower *Pomc* and *Cart* compared to controls, with HFD *Bace1* ^*KO*^ mice retaining higher *Npy* and *Agrp* and lower *Pomc* levels than HFD control mice. Reduction of Bace1 activity by M-3 in DIO mice increased the neuropeptide transcript ratio, reversing the HFD-mediated suppression of orexigenic and/or increase in anorexigenic neuropeptides, respectively. Consequently, raised Bace1 levels and activity by HFD may contribute to the failure of ARC neurons to respond normally to orexigenic cues associated with fasting.

DIO mice exhibit leptin resistance, whereby exogenous leptin is unable to reduce food intake or body weight, whereas HFD *Bace1* ^*het*^ and *Bace1* ^*KO*^ mice retain sensitivity to exogenous leptin. As altered Bace1 activity had little or no effect on daily food intake, the increased leptin efficacy probably resides in hypothalamic circuits that govern energy expenditure and thermogenesis^32^. Because the hypophagia induced by MTII was unaffected in either dietary regime by Bace1 expression status, the altered leptin responsiveness is likely centred on ARC first order neurons, a notion supported by unchanged second order hypothalamic neuron neuropeptide transcription levels by fasting or leptin in *Bace1* ^*KO*^ mice. Interestingly, leptin increased *Pomc* and *Cart*, with *Npy* and *Agrp* unaffected, in HFD *Bace1* ^*KO*^ mice. Thus, in the absence of Bace1, the ARC neuropeptide transcriptional programme retains selective leptin sensitivity with POMC neurons being less susceptible to the effects of caloric excess than NPY/AgRP neurons.

Inhibition of Bace1 activity by M-3 or AZ-4217 in DIO mice lowered hypothalamic Bace1 levels and activity. This was associated with decreased body weight, fat mass, plasma leptin and improved glucose homeostasis, with no reduction in food intake or lean mass, emulating genetic Bace1 reduction^6^. In contrast, RCD control mice were refractory to M-3, showing a mildly improved peripheral glucose disposal, perhaps indicative of increased muscle glucose uptake^6,33^. Thus, metabolic responsiveness to pharmacologically driven reduced Bace1 activity is primarily observed in obese, hyperleptinemic and leptin resistant mice, where hypothalamic Bace1 protein content and activity have been increased above lean control levels by chronic HFD. The ability of Bace1 inhibition to improve the metabolic phenotype of obese mice requires intact leptin signalling competence, as denoted by minimal effects of M-3 on *ob/ob* and *db/db* mice. Furthermore, inhibition of Bace1 activity in *ob/ob* and *db/db* mice did not affect glucose homeostasis, indicating that the improved glucose disposal of DIO mice is also dependent on recovery of leptin sensitivity. These data suggested the notion that reducing Bace1 activity in DIO mice re-sensitizes hypothalamic circuits to endogenous leptin. However, in lean RCD mice, M-3 did not potentiate endogenous or exogenous leptin action compared to vehicle controls. In contrast, M-3 reduced body weight at high levels of endogenous leptin and reduced food intake and body weight in response to exogenous leptin in leptin resistant DIO mice. Increased leptin sensitivity was also observed at the biochemical level, with M-3 augmenting the hypothalamic pSTAT3^Tyr705^ response to exogenous leptin in DIO leptin resistant, but not lean RCD mice. Collectively, these results indicate that reducing Bace1 activity restores leptin sensitivity in a DIO leptin resistant state, but does not sensitize hypothalamic circuits to the low levels of endogenous leptin in lean mice.

First, we sought an explanation for retention of leptin responsiveness in *Bace1* ^*KO*^ mice and recovery of leptin sensitivity in M-3 treated DIO mice by examining negative regulators of leptin signalling. Raised hypothalamic pSTAT3^Tyr705^ promotes the transcription and accumulation of *Socs3*, which attenuates leptin signalling^34,35^. *Socs3* is increased by exogenous leptin in RCD control and *Bace1* ^*KO*^ mice and in DIO mice basal *Socs3* is higher and the leptin response blunted, whereas HFD *Bace1* ^*KO*^ mice exhibit normal basal and leptin-stimulated *Socs3*. It is likely that the increased basal pSTAT3^Tyr705^ and *Socs3* and SOCS3 levels in DIO mice are driven by the endogenous high leptin levels, contrasting with HFD *Bace1* ^*KO*^ mice, which have low levels of endogenous leptin and so reduced basal pSTAT3^Tyr705^ and SOCS3. DIO mice have raised basal pSTAT3^Tyr705^ with no further increase elicited by exogenous leptin, whereas RCD and HFD *Bace1* ^*KO*^ mice have lower basal pSTAT3^Tyr705^ and exhibit a robust response to leptin. Previous studies have shown that DIO-driven leptin resistance elevates basal ARC pSTAT3^Tyr705^ and reduces its response amplitude to exogenous leptin^11,36,37^. Thus, our data are consistent with the idea that increased baseline hypothalamic pSTAT3^Tyr705^ and SOCS3 in DIO mice are the result of ongoing endogenous leptin action^38^. Consequently, we considered the possibility that another factor dependent on Bace1 activity, additional to the hyperleptinemia in the obese state, was responsible for opposing leptin physiological responses in DIO mice.

As the predominant outcome of Bace1 activity is cleavage of APP and Aβ production, we focused on this pathway. HFD raised hypothalamic Aβ_1–42_, but not the more prevalent Aβ_1–40_, with the increase only observed in late stage HFD challenge. Consequently, we surmise that the dietary challenge modifies γ-secretase activity to enable a selective increase in Aβ_1–42_ over Aβ_1–40_. Although inhibition of Bace1 with M-3 or AZ-4217 lowered hypothalamic Aβ peptide levels in all obese mice, this was insufficient to lower body weight and recover glucose homeostasis in the absence of leptin signalling, as shown by in *ob/ob* and *db/db* mice. Raising brain Aβ_1–42_ in lean mice induced a small, but significant, increase in body weight, whereas mice on a HFD were more susceptible to the obesogenic action of central Aβ_1–42_. Indeed, raised hypothalamic Aβ_1–42_ induced induction of leptin resistance in early-stage HFD mice, as demonstrated by loss of responsiveness to exogenous leptin at the physiological (body weight and food intake) and cellular (reduced amplitude of pSTAT3^Tyr705^) levels. Furthermore, even in RCD WT and *Bace1* ^*KO*^ mice, raised central Aβ_1–42_ for 21 days was sufficient to reduce hypothalamic leptin sensitivity. The mechanism(s) underlying the induction of leptin resistance by Aβ_1–42_ appear not to be directly linked to increased hypothalamic levels of PTP1B or SOCS3, or to the presence of ER stress or inflammation. However, raised hypothalamic Aβ_1–42_ was associated with increased basal pSTAT3^Tyr705^, perhaps reflecting increased plasma leptin, resulting in diminished pSTAT3^Tyr705^ signal amplitude in response to exogenous leptin, mimicking a key impairment associated with the phenomenon of dietary-induced leptin resistance (Supplementary Fig. 8).

As hypothalamic Aβ_1–42_ levels are unaffected in early stage HFD challenge when there is a lack of inflammation or raised PTP1B or SOCS3, we suggest that late-stage chronic HFD engages an inflammatory response in the hypothalamus, which jointly drive higher Bace1 activity and increased production of Aβ_1–42_, leading to further pro-inflammatory signalling through induction of a vicious cycle^39^. Furthermore, the increased hypothalamic cytokine and Aβ_1–42_ levels would provide an additional stimulus, along with hyperleptinemia, to increase SOCS3 and PTP1B.

In summary, we have demonstrated that raised hypothalamic Aβ_1–42_, in conjunction with HFD, is sufficient to induce leptin resistance in the absence of increased inflammation, ER stress, SOCS3 and PTP1B. Thus, counteracting raised Bace1 activity associated with chronic HFD will play a critical protective role against metabolic disease and suggest that pharmacological approaches, including repurposing of Bace1 inhibitors currently being developed for AD, represent a novel strategy for the treatment of obesity and T2D.

## Methods

### Mice

All mice given free access to food and water and maintained on a 12 hr light/dark cycle. All animal care, experimental protocols and procedures performed in accordance to the Animal Scientific Procedures Act (1986), with approval of Universities of Dundee and Aberdeen Ethics Committees. NPY-hrGFP (Jackson Laboratory), POMC-eGFP^8^ and GAD67-GFP, VGlut2-GFP and LEPR-GFP mice on a C57BL/6 background were used. *Bace1* transgenic mice on the C57Bl6/J background^6^ provided *Bace1* ^*KO*^, *Bace1* ^*het*^ and wild-type control littermates. Male mice, homozygote for leptin, *ob/ob* (B6.V-Lep^ob^/OlaHsd) or leptin receptor, *db/db* (BKS.Cg- + Lepr^db^/+ Lepr^db^/OlaHsd) mutations were obtained from Harlan Laboratories. Male (all on C57BL6/J background) mice were fed regular chow (RC: 4% calories from fat: RMI 505) throughout or at 8–10 weeks of age switched to HF diet (45% calories from fat: SDS 824053) for 20 weeks. Food intake, body weight, fat mass, glucose tolerance, insulin sensitivity and serum endocrine and biochemical parameters were measured as described in ref.^6^. Leptin sensitivity was assessed by: (i) reduction in body weight and food intake in response to i.p leptin (2 mg/kg twice daily for 3 days) monitored *in vivo* and (ii) *ex vivo* quantification, by RT-qPCR and immunoblotting of BMH mRNA and protein levels, in response to a single i.p. dose of leptin (3 mg/kg). For acute M-3 exposure, mice received twice daily i.p. saline for 3 days to become conditioned to the procedure. Subsequently, mice received M-3 (10 mg/kg) or vehicle daily by i.p for 5 days, with the final three days also receiving leptin twice daily (2 mg/kg) or saline. MTII sensitivity determined after a single i.p. dose of MTII (50 μg) with food intake measured over the subsequent 24 hours. Respiratory exchange ratios and activity were monitored using a comprehensive lab animal monitoring system (Columbus Instruments, Columbus, OH, USA).

### Intraperitoneal injections

Leptin and MTII were dissolved in phosphate-buffered saline (PBS) and Merck-3 (β-secretase inhibitor IV, Calbiochem) dissolved in 50:50 DMSO:PBS. Agents administered intraperitoneally as a single dose (MTII, 50 µg; leptin, 3 mg/kg), once daily for 5 days (M-3, 10 mg/kg) or twice daily for 3 days (leptin, 2 mg/kg) in independent cohorts of mice. Body weight and food intake were monitored daily.

### Osmotic minipump drug delivery

Osmotic minipumps (2004 model, Alzet) were implanted under isoflurane anesthesia and delivered M-3 (10 mg/kg/day), vehicle (50:50 DMSO:PBS), Aβ_1–42_ or ScrP (3.36 µg/kg/day or 10 µg/kg/day) for 14, 21 or 28 days to DIO or *ob/ob* and *db/db*, or control mice. Food intake and body weight were measured from 3–4 days after implant to allow stabilisation. Peripheral administration was by subcutaneous implantation. Central administration was achieved by osmotic minipump connected to a catheter implanted into the lateral ventricle. Food intake and body weight were measured from 3–4 days after implant to allow stabilisation. AZ-4217 (50 mg/kg/day) or vehicle (0.3 M Gluconic acid, pH 3) administered by oral gavage to DIO mice for 4 weeks.

### Gene expression analysis and immunoblotting

RNA was extracted from BMH wedges or hypothalamic sections as described^6^ and gene expression analysed using commercially available gene-specific Taqman probes with primer sets (Supplementary Table S1). Xbp1 mRNA was measured using SYBR Green-based qPCR (*Xpb1s* Fw CTGAGTCCGAATCAGGTGCAG; Rev GTCCATGGGAAGATGTTCTGG and *Xbp1u* Fw CAGCACTCAGACTATGTGCA; Rev GTCCATGGGAAGATGTTCTGG). For immunoblotting, BMH or hypothalamic levels of total and phosphorylated proteins were separated by SDS-PAGE and transferred to nitrocellulose membranes^6^, with detection by commercially antibodies (Supplementary Table S2) and chemiluminescence with quantification by standard imaging techniques. Bace1 levels were measured using a Sensizyme Bace1 activity assay (Sigma).

### ELISAs

Aβ_1–40_, Aβ_1–42_, sAPPβ, inflammatory cytokines (MesoScaleDiscovery) and STAT3(pY^705^) (Invitrogen) were measured in hypothalamic lysates (50 µg) following manufacturer instructions.

### Brain immunohistochemistry

Bace1 immunohistochemistry performed on hypothalamic slices from Control, POMC-eGFP, NPY-GFP, GAD67-GFP, VGlut2-GFP and LEPR-GFP mice. Brains were fixed in 4% paraformaldehyde in phosphate buffer solution (PBS) for 24 hours at 4 °C followed by post-fixing in 30% sucrose in PBS. Tissue was cut into 30 µm sections on a cryostat and free-floating sections collected serially in PBS. Sections were permeabilised in PBS-T (PBS + 0.1% Triton X-100) for one hour, then blocking for one hour (5% BSA in PBS-T) at room temperature. Following PBS-T rinse sections were incubated with primary antibodies (see Supplemental Table S2) and agitated overnight at 4 °C. Following removal of primary antibody, sections were washed in PBS-T (5 × 5 minutes) before incubation in the appropriate secondary antibody, for one hour at room temperature. Following removal of the secondary antibody sections were washed in PBS-T (5 × 5 minutes) to remove any remaining Triton. Sections were mounted on glass microscope slides (VWR International) with Vectashield anti-fade medium (Vector Laboratories). Coverslips were placed over the sections, sealed with nail varnish and stored at 4 °C until imaged. For co-staining experiments, sequential staining was carried out, whereby following primary antibody removal and washes, a second primary antibody was added with a further overnight incubation step. The same method was adopted for secondary antibody incubations steps. Fluorescent images were acquired using a confocal laser-scanning microscope (Leica TCS SP5 II) with x 40 oil-objective lens and LAS software. Controls were carried out where primary antibodies where omitted with minimal fluorescence observed. Images were processed using Volocity Version 6.3 (Perkin & Elmer) and Adobe Photoshop CC 2015. This involved merging Z-stack single-plane images to give maximum projection images (as representatives) and acquiring HD snapshots and exporting as TIFF files. Scalebars were added and for some images, where necessary, brightness and contrast was adjusted and background noise reduced (using the “remove noise” function on Volocity) before cropping. For cell counting experiments images were analyzed using ImageJ64. Maximum projection TIFF images were used in all cases, as representatives, and cells were manually counted using the Cell Counter plugin. A cell was counted as ‘co-localized’ where both cells in the individual channels overlapped and this was confirmed by viewing single-plane images from the corresponding Z-stacks.

### Fluorescent *In situ* hybridization (FISH)

Fluorescent *in situ* hybridization for *POMC* and *NPY* performed on hypothalamic brain sections from control mice. Primer sequences for POMC (151–850 bp, Genbank: NM_008895.4) and NPY (104–501 bp, Genbank: NM_023456.2) were chosen, amplified and cloned in the pBluescript II SK(−) plasmid. To generate antisense cRNA probes, plasmids were linearised with a BamHI restriction enzyme for 1.5 hours at 37 °C. To confirm digestion worked correctly and the products were the correct size, linearised plasmids were run on a 1% agarose gel in TBC buffer. Linearised DNA was purified using a PCR purification kit (Qiagen) following their protocol. To ensure purification worked correctly purified DNA was run on a 1% agarose gel. The concentration of purified DNA was measured using a Nanodrop spectrophotometer (Thermo Scientific). *In vitro* transcription (IVT) was carried out for two hours at 37 °C using Digoxigenin (DIG)- labeling mix, RNase inhibitor, buffer, DTT and polymerase. After IVT the template DNA was cut using DNase I and RNase and incubated for one hour at 37 °C. RNA was purified using Purelink RNA mini kit (Ambion Life Technologies) for ISH. Lysis buffer was added to samples to protect the RNA from degrading, followed by RNA purification. Purified RNA was run on a 1% agarose gel to ensure the IVT and purification was successful. Probes were stored at −20 °C

Mice were perfused fixed using 4% paraformaldehyde (PFA) in PBS with 2 mM EGTA and brains removed for post-fixing overnight at 4 °C. Brains were washed (2 × 10 minutes) in PBS-T (PBS + 0.1% Tween-20) rotating before freezing in cryo-embedding media in preparation for frozen cryo-sectioning. Whole brains were sectioned at 30 μm by cryostat and collected free-floating in 50% ethanol (ETOH) in PBST. Frozen sections were washed in 50% ETOH/PBST for 10 minutes, followed by 100% ETOH washes (2 × 10 minutes) rotating, before storage at −20° in 100% ETOH. Sections were allowed to come to room temperature before rehydrating in 50% ETOH/PBST for 10 minutes, followed by PBS-T washes (2 × 10 minutes) on a rocking platform then treated with proteinase K (10 μg/ml) in PBST for 8 minutes. Proteinase K was removed and sections rinsed in PBS-T before post-fixing for 30 minutes in 4% HCHO + 0.1% glutaraldehyde in PBS-T on a rocking platform, then washed (2 × 10 minutes) in PBS-T and then in pre-warmed 1:1 PBS-T/exonic hybridization mix solution for 10 minutes at 65 °C. Sections were rinsed twice in warmed exonic hybridization mix and allowed to settle before incubation in fresh warmed exonic hybridization mix for 2 hours at 65 °C. Sections were then incubated in pre-warmed exonic hybridization mix with DIG-labeled probes (80μl/ml) overnight at 65 °C horizontally.

Probes were recovered and stored at −20 °C, sections rinsed twice with pre-warmed exonic hybridization mix, before washes (3 × 1 hour) in pre-warmed exonic hybridization mix at 65 °C. Sections were washed (1 × 15 minutes) in a pre-warmed 1:1 TBS-T (1x tris-buffered saline + 0.1% Tween-20)/exonic hybridization mix solution at 65 °C, brought to room temperature and rinsed thoroughly with TBS-T for 30 minutes on a rocking platform, before blocking and antibody incubation steps. Blocking was carried out for 2 hours at room temperature in TBS-T with 2% blocking buffer reagent and 20% heat-treated goat serum solution. After pre-incubation, sections were incubated in anti-DIG-HRP antibody in blocking solution (1:200) overnight at 4 °C on a rocking platform. Sections were rinsed three times in TBS-T at room temperature before washing (3 × 1 hour) with TBS-T. Sections were then transferred to eppendorf tubes and incubated in amplification buffer (Cy3-TSA kit, Perkin & Elmer) for 1 minute, followed by incubation in Cy3-TSA solution (Cy3-TSA kit, Perkin & Elmer) for 90 minutes, in the dark. TSA-solution was discarded and sections washed (3 × 5 minutes) in TBS-T and transferred back into wells, incubated in 1% H_2_O_2_ in TBS-T for 45 minutes at room temperature and washed thoroughly in TBS-T (3 × 5 minutes) and PBS-T (2 × 5 minutes). Sections were processed for immunohistochemistry as previously described. Fluorescent images acquired using a laser scanning confocal microscope (Leica TCS SP5 II). In all cases Z-stack images were taken at either x 40 or x 63 magnification using an oil-objective lens, on LAS software.

### Statistical Analysis

Data are means ± SEM and analysed using GraphPad Prism version 6 (GraphPad Software). For comparisons between two groups, 2-tailed unpaired Student’s t-tests were used and for multiple groups, one- or two-way ANOVA, with or without repeated measures, followed by an appropriate multiple comparison post-test. *P* < 0.05 was considered significant.

### Data availability statement

The datasets generated during and/or analysed during the current study are available from the corresponding author on reasonable request.

## Electronic supplementary material

Supplementary Information
