# Supplementary material for: Bace1-dependent amyloid processing regulates hypothalamic leptin sensitivity in obese mice
Source: Sci Rep. 2018 Jan 8;8:55. doi: 10.1038/s41598-017-18388-6 (PMC5758523; doi:10.1038/s41598-017-18388-6)
Supplement: Supplementary file 1 — Supplementary Information [file 41598_2017_18388_MOESM1_ESM.pdf]

Bace1-dependent amyloid processing regulates hypothalamic leptin sensitivity in obese mice

Paul J. Meakin[1], Susan M. Jalicy[1], Gemma Montagut[1], David J.P. Allsop[1], Daniella L. Cavellini[1], Stuart W. Irvine[1], Christopher McGinley, Mary K Liddell[1], Alison D McNeilly[1], Karolina Parmionova[1], Yu-Ru Liu[2], Charlotte L. S. Bailey[2], J. Kim Dale[2], Lora K Heisler[3], Rory J McCrimmon[1] & Michael L. J. Ashford[1*]

[1]Division of Molecular and Clinical Medicine, School of Medicine, Ninewells Hospital & Medical School, Dundee DD1 9SY, UK.

[2]School of Life Sciences, University of Dundee, Dundee DD1 5EH, UK

[3]Rowett Institute of Nutrition and Health, Aberdeen AB21 9SB, UK

*Correspondence:

Michael LJ Ashford

Division of Molecular and Clinical Medicine

School of Medicine, University of Dundee

Ninewells Hospital & Medical School

Dundee DD1 9SY

Tel: +44 1382 383095

Email: m.l.j.ashford@dundee.ac.uk

Supplementary Table 1. qRT-PCR probes and primers used in the study

Probe Name	Code
Actin	4352933E
AgRP	Mm004475829_g1
Atf4	Mm00515325_g1
Atf6	Mm01295319_m1
Bace1	Mm00478664_m1
Cartpt	Mm0048906_m1
Crh	Mm01293920_s1
Ddit3	Mm01135937_g1
Gadd34	Mm01205601_g1
Hcrt	Mm0196403_s1
Hspa5	Mm00517690_g1
Lepr	Mm00440181_m1
Mc4r	Mm00457483_s1
NPY	Mm03048253_m1
Pmch	Mm01242886_g1
POMC	Mm00435874_m1
PTP1B	Mm00448431_m1
SOCS3	Mm00545913_s1
Trh	Mm01963590_s1

Supplementary Table 2. Primary and Secondary antibodies used in the study

Primary Antibody	Species	Dilution	Source
Bace1	Rabbit	1:250 (IHC)<br>1:1000 (IB)	Sigma Aldrich<br>(B0681)
NeuN	Guinea-Pig	1:500 (IHC)	Millipore<br>(ABN90P)
LEPR-B	Sheep	1:100 (IHC)	In-house
pSTAT3<br>(Tyr 705)	Rabbit	1:1000 (IB)<br>1:500 (IB)	Cell Signalling<br>(9131)
STAT3	Mouse	1:1000 (IB)	Cell Signalling<br>(9139)
GFP	Mouse	1:200 (IHC)	Abcam<br>(ab291)
GFP	Chicken	1:200 (IHC)	Abcam<br>(ab13970)
GFAP	Chicken	1:500 (IHC)	Millipore<br>(AB5541)
Actin	Rabbit	1:5000 (IB)	Sigma Aldrich<br>(A2066)
PTP1B	Rabbit	1:1000 (IB)	Dr M Delibegovic<br>(U of Aberdeen)
Mitofusin 1	Mouse	1:1000 (IB)	Abcam<br>(ab126575)
Mitofusin 2	Mouse	1:1000 (IB)	Abcam<br>(ab56889)
p-EIF2α	Rabbit	1:1000 (IB)	Cell Signalling<br>(3398)
EIF2α	Rabbit	1:1000 (IB)	Cell Signalling<br>(5324)
SOCS3	Rabbit	1:1000 (IB)	Cell Signalling<br>(2923)
p-IRE1	Rabbit	1:1000 (IB)	Abcam<br>(ab48187)
IRE1	Rabbit	1:1000 (IB)	Abcam<br>(ab37073)
CHOP	Rabbit	1:1000 (IB)	Cell Signalling<br>(5554)

Secondary Antibody	Dilution	Source
Alexa 488	1:500 (IHC)	Life Technologies
Alexa 647	1:500 (IHC)	Life Technologies
Cy3	1:250 (IHC)	Jackson ImmunoResearch

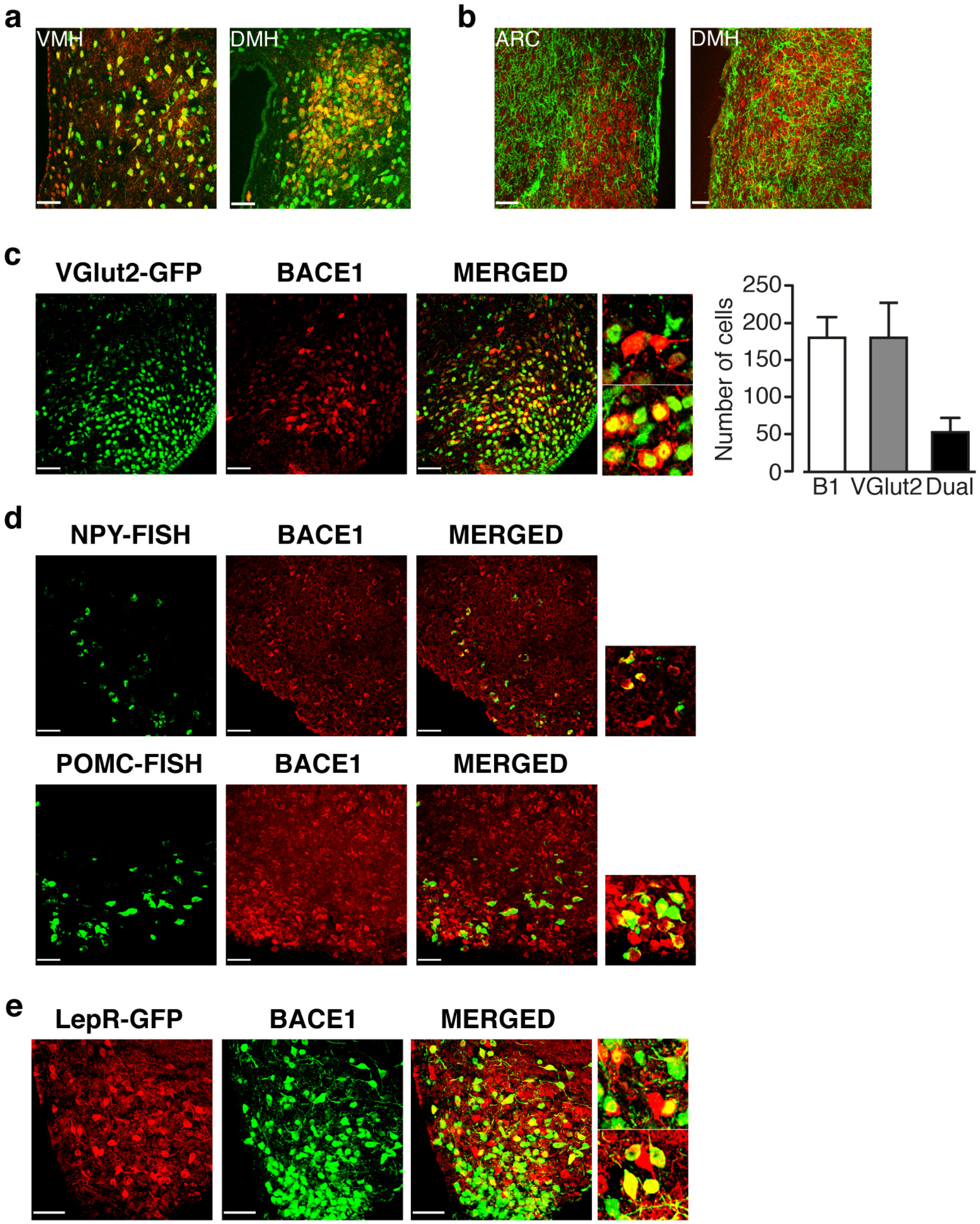

Supplementary Figure 1. Hypothalamic neuronal expression of Bace1.
(**a**) Representative photomicrograph of VMH and DMH Bace1(red) and NeuN (green) immunoreactivity showing co-localisation. Bar, 50 μm. (**b**) Representative photomicrograph of ARC and DMH Bace1 and GFAP immunoreactivity showing absence of co-localisation. Bar, 50 μm. (**c**) Bace1 immunoreactivity in ARC of VGlut2-GFP mice, with merged images (with inset higher magnification) showing dual-label immunofluorescence. Bars, 50 μm. (**d**) Representative photomicrographs of Bace1 immunoreactivity and POMC and NPY mRNA fluorescent in situ hybridization. Bar 50 μm. (**e**) Bace1 immunoreactivity in ARC of LepR-GFP mice, with merged images showing dual-label immunofluorescence. Bars, 50 μm. Bar graph (**c**) shows number of cells singly- and dual-labelled from 3 different mice.

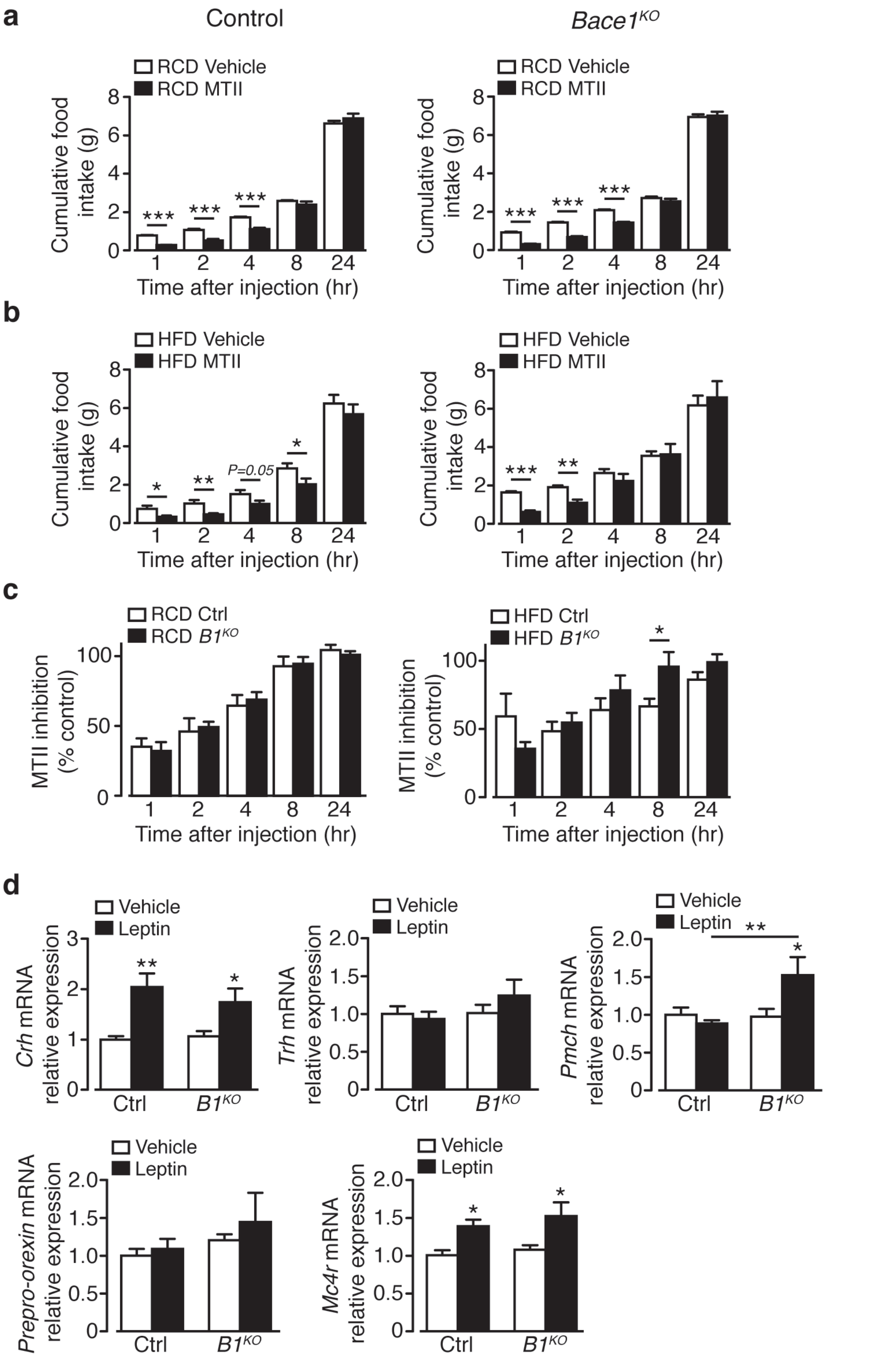

Supplementary Figure 2. Melanotan II-mediated hypophagia and neuropeptide expression of second order hypothalamic neurons is unaltered in *Bace1^{KO}* mice. Cumulative food intake at the times indicated after injection of vehicle or MTII following an overnight fast in (**a**) RCD control and *Bace1^{KO}* mice (n = 7/group) and (**b**) HFD (20 weeks) control and *Bace1^{KO}* mice (n = 7/group). (**c**) Relative inhibition of feeding by MTII at the times indicated after injection in RCD and HFD control and *Bace1^{KO}* mice (**d**) RT-qPCR analysis of *Crh*, *Trh*, *Pmch*, Prepro-orexin and *Mc4r* from vehicle or leptin-treated RCD control and *Bace1^{KO}* mice (n = 7 – 9/group). Data normalized to vehicle treated control mice. Data are means ± SEM. * $P < 0.05$, ** $P < 0.01$, *** $P < 0.001$ by one-way ANOVA with Bonferroni's multiple comparisons test (**a** – **c**) or Kruskal-Wallis test (**d**).

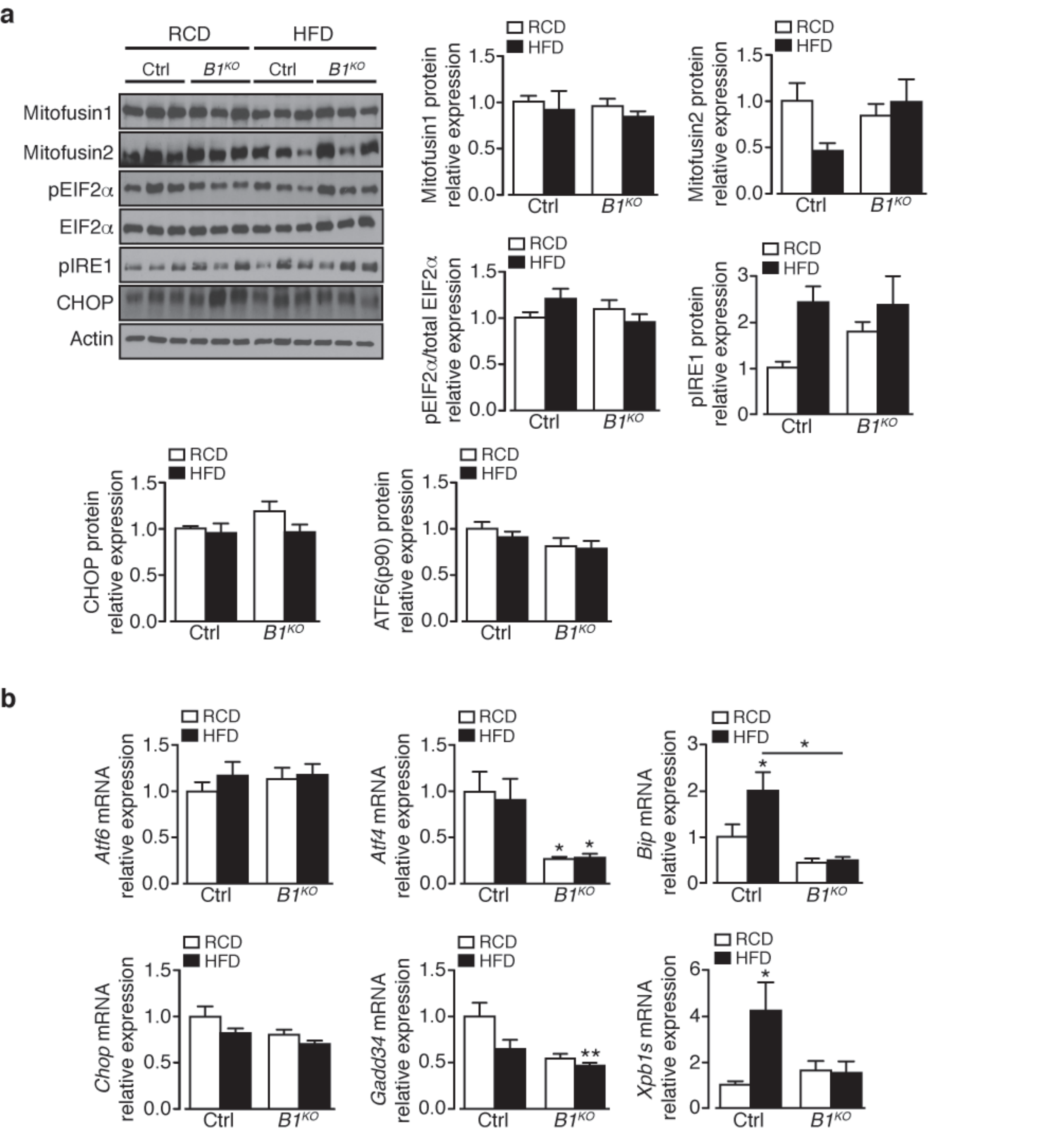

Supplementary Figure 3. Effect of lack of Bace1 on hypothalamic ER stress markers. (**a**) Immunoblots of mitofusin1, mitofusin2, pEIF2α & EIF2α, pIRE1, CHOP and actin in BMH of control and *Bace1*^{KO} mice on RCD and HFD. The quantified signal intensity of each protein is shown (*n* = 6 – 9/group). (**b**) RT-qPCR analysis of BMH *Atf6*, *Atf4*, *Bip*, *Chop*, *Gadd34*, and *Xbp1s* from RCD and HFD control and *Bace1*^{KO} mice (*n* = 6 - 9/group). In each case data are normalized to RCD controls. Data are means ± SEM. * P < 0.05, ** P < 0.01 by Kruskal-Wallis test. Uncropped images of immunoblots can be found in Supplementary Fig. 12.

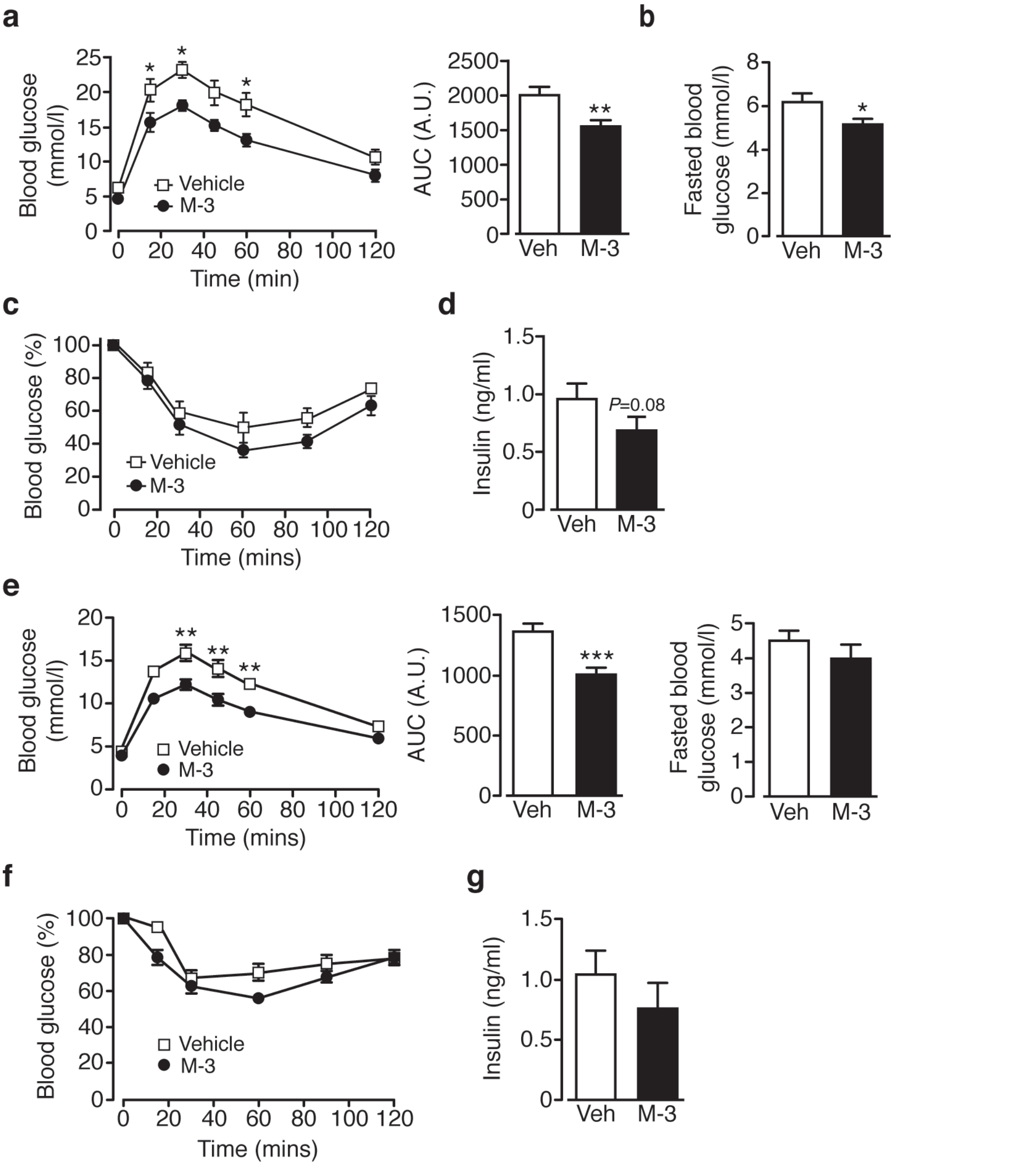

Supplementary Figure 4. Peripheral or central administration of Bace1 inhibitor improves glucose homeostasis in DIO mice.

(**a**,**b**) Glucose tolerance (with AUC) after 14 days sc M-3 (**a**) and fasted glucose (**b**) after 24 days M-3 in DIO mice (*n* = 9/group). (**c**,**d**) Insulin sensitivity (**c**) and fasted serum insulin (**d**) levels after 24 days sc M-3 in DIO mice (*n* = 7 - 10/group). (**e**) Glucose tolerance (with AUC) after 14 days icv M-3 (*n* = 11/group). (**f**,**g**) Insulin sensitivity (**f**) and fasted serum insulin (**g**) levels after 14 days icv M-3 in DIO mice (*n* = 11 –14/group). Data are means ± SEM. * P < 0.05, ** P < 0.01, *** P < 0.001 by repeated measures ANOVA with Sidak's multiple comparisons test (**a**,**c**,**e**,**f**) or 2-tailed unpaired Student's t-test (**b**,**e**,**g** and AUC in **a**,**e**)

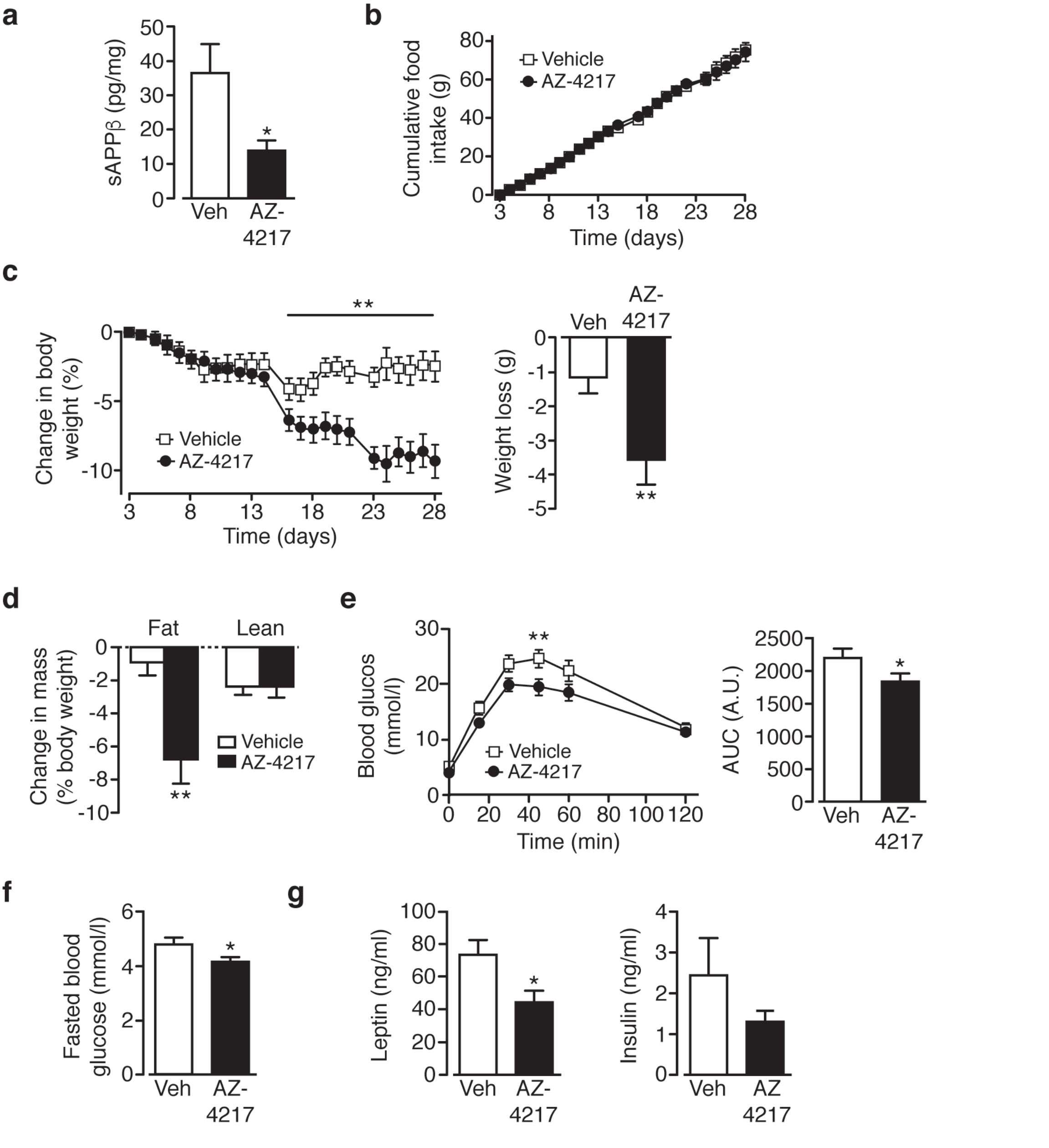

Supplementary Figure 5. Oral administration of AZ-4217 improves metabolic phenotype of DIO mice.

(**a**) BMH Bace1 activity in vehicle ($n = 9$) or AZ-4217 treated DIO mice ($n = 10$). (**b**) Cumulative food intake of age-matched DIO mice treated with vehicle ($n = 9$) or AZ-4217 ($n = 10$). (**c**) Percentage decrease in body weight and actual body weight loss in age-matched DIO mice treated with vehicle ($n = 9$) or AZ-4217 (n =10). (**d**) qMR scans showing fat and lean mass in DIO mice treated with vehicle ($n = 9$) or AZ-4217 (n =10) for 28 days. (**e**) Glucose tolerance (with AUC) after 14 days and (**f**) fasted plasma glucose levels after 24 days, vehicle ($n = 9$) or AZ-4217 ($n = 10$) in DIO mice. (**g**) Plasma leptin and insulin levels after 28 days treatment with vehicle ($n = 9$) or AZ-4217 ($n = 10$) in DIO mice. Data are means ± SEM. * $P < 0.05$, ** $P < 0.01$ by repeated measures ANOVA with Sidak's multiple comparisons test (**b**,**c**,**e**).1-way ANOVA with Bonferroni's multiple comparisons test (**d**) or 2-tailed unpaired Student's t-test (**a**,**f**,**g**, Body weight loss in **c** and AUC in **e**).

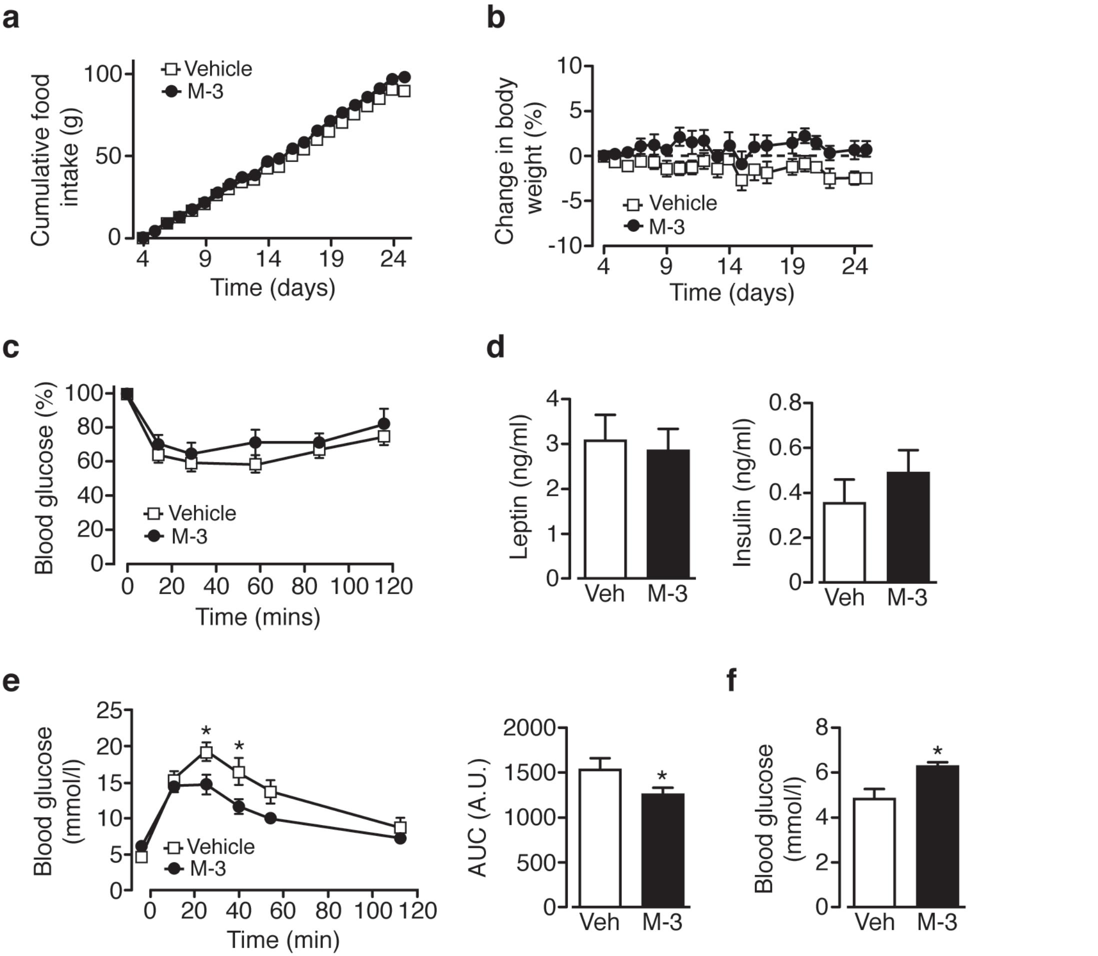

Supplementary Figure 6. M-3 has minor effects on the metabolic phenotype of lean mice.

(**a**) Cumulative daily food intake of control mice (*n* = 7/group). M-3 had no effect on body weight (**b**), insulin sensitivity (**c**) or serum leptin and insulin levels (**d**) compared to vehicle in RCD control mice (*n* = 7/group). (**e**,**f**) Glucose tolerance (with AUC) after 14 days and fasted blood glucose after 24 days, M-3 vs vehicle in control mice (*n* = 7/group). Data are means ± SEM. * $P < 0.05$ by repeated measures ANOVA with Sidak's multiple comparisons test (**a**,**b**,**c**,**e**) or 2-tailed unpaired Student's t-test (**d**,**f** and AUC in **e**).

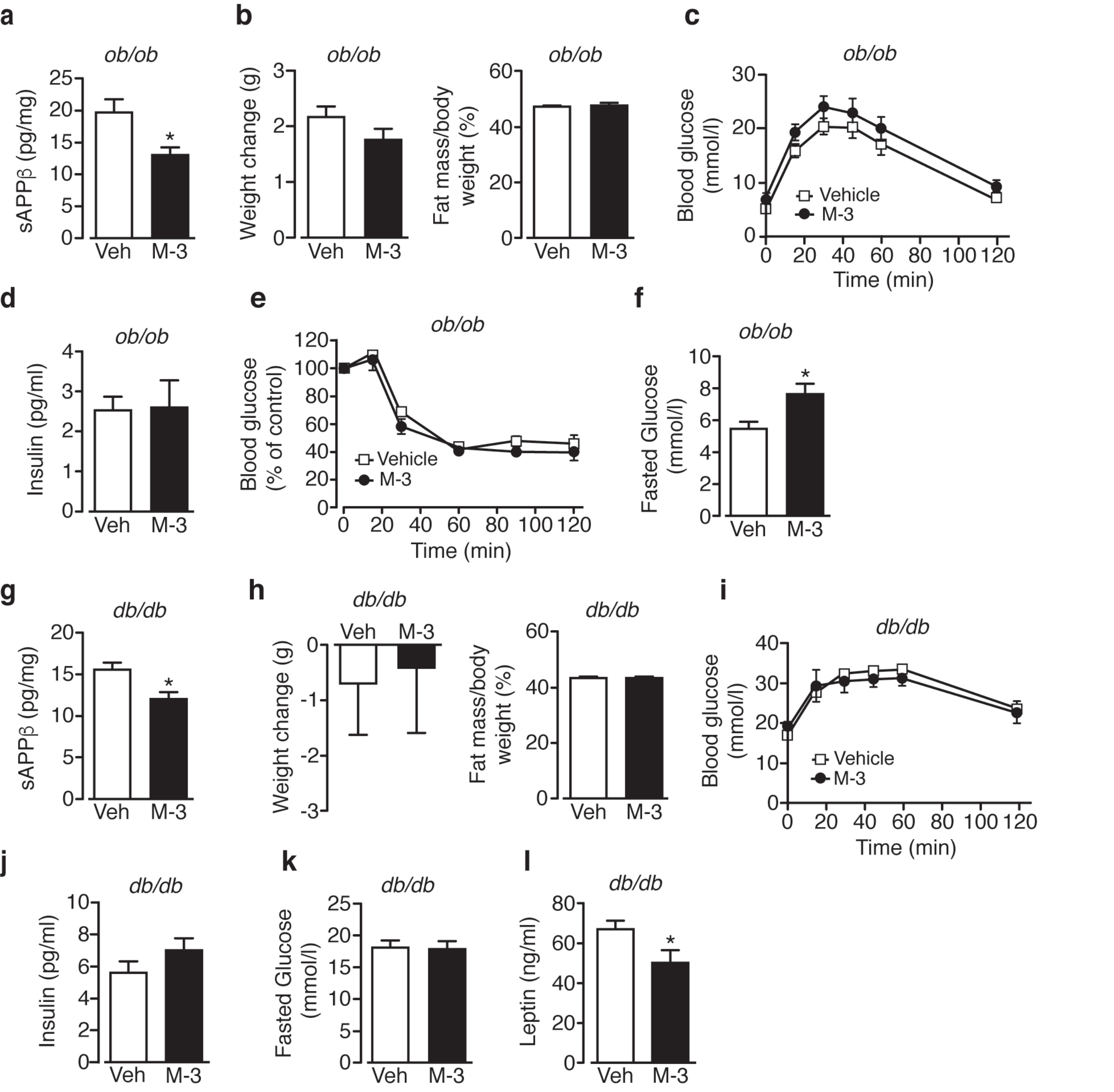

Supplementary Figure 7. M-3 has no effect on fat or glucose homeostasis in *ob/ob* or *db/db* mice.

(**a**) BMH Bace1 activity (sAPPβ levels) in sc vehicle ($n = 8$) or M-3 ($n = 6$) treated *ob/ob* mice. (**b**) Actual body weight change and % fat mass in *ob/ob* mice sc treated with vehicle ($n = 11$) or M-3 ($n = 10$). Glucose tolerance (**c**), fasted serum insulin (**d**), insulin sensitivity (**e**) and fasted blood glucose (**f**) of *ob/ob* mice sc treated with vehicle or M-3 ($n = 10 - 11$/group). (**g**) BMH Bace1 activity (sAPPβ levels) in sc vehicle ($n = 5$) or M-3 ($n = 4$) treated *db/db* mice. (**h**) Actual body weight change and % fat mass in *db/db* mice sc treated with vehicle ($n = 9$) or M-3 ($n = 8$). Glucose tolerance (**i**), fasted serum insulin (**j**), fasted blood glucose (**k**) and plasma leptin (**l**) levels of *db/db* mice sc treated with vehicle or M-3 ($n = 8 - 10$/group). Data are means ± SEM. * $P < 0.05$ by 2-tailed unpaired Student's t-test (**a**,**b**,**d**,**f**,**g**,**h**,**j**,**k**,**l**) or repeated measures ANOVA with Didak's multiple comparisons test (**c**,**e**,**i**).

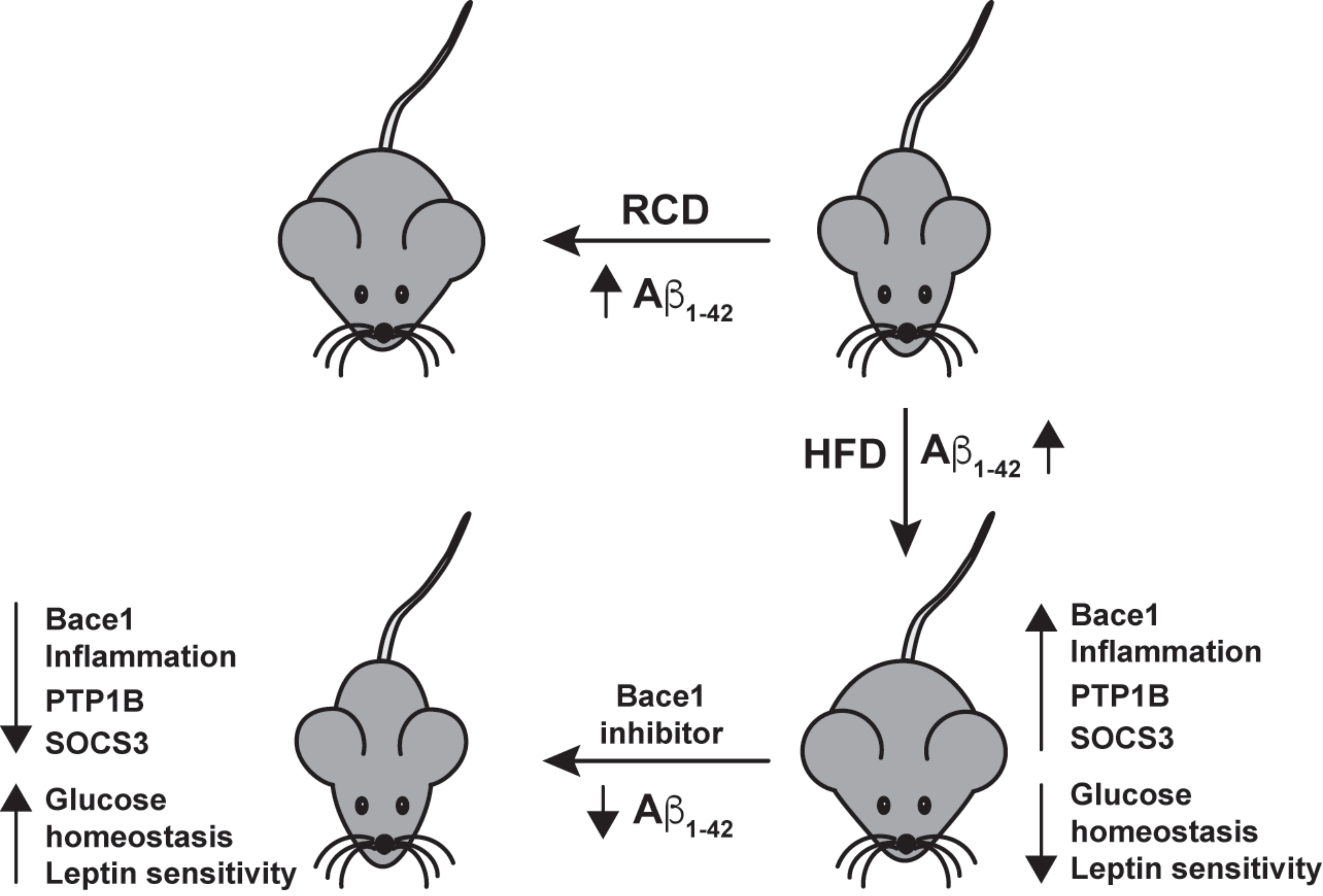

Supplementary Figure 8: Model showing how altered central $A\beta_{1\text{-}42}$ levels are related to Bace1 activity and to changes in hypothalamic inflammation, regulation of hypothalamic leptin sensitivity, body weight and glucose homeostasis.

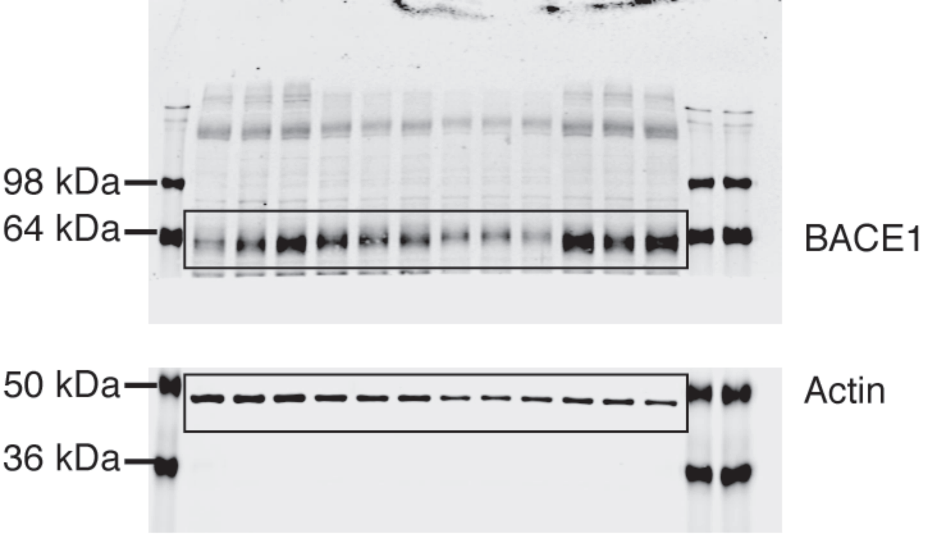

Supplementary Figure 9. Immunoblots that illustrate the presence of Bace1 in mouse brain regions. These are uncropped immunoblot images corresponding to data shown in Figure 1b.

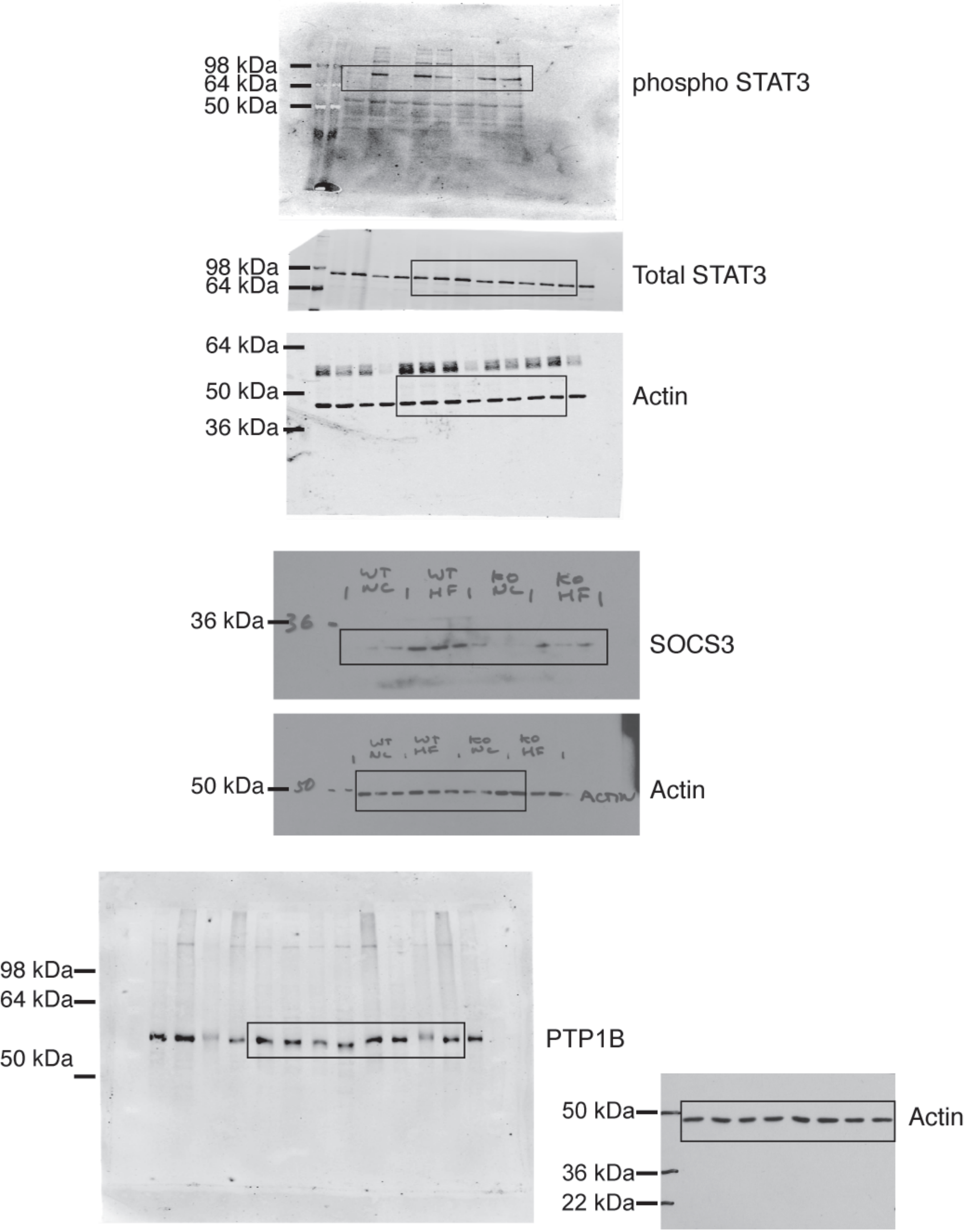

Supplementary Figure 10. Immunoblots that illustrate how the absence of Bace1 alters p-STAT3, SOCS3 and PTP1B levels in response to leptin and/or diet. These are uncropped immunoblot images corresponding to data shown in Figures 3a, 3g and 3h, respectively. The PTP1B antibody has been characterised previously [Thompson D, Morrice N, Grant L, Le Sommer S, Lees EK, Mody N, Wilson HM, Delibegovic M. Clin Sci (Lond). 2017;131(20):2489-2501]

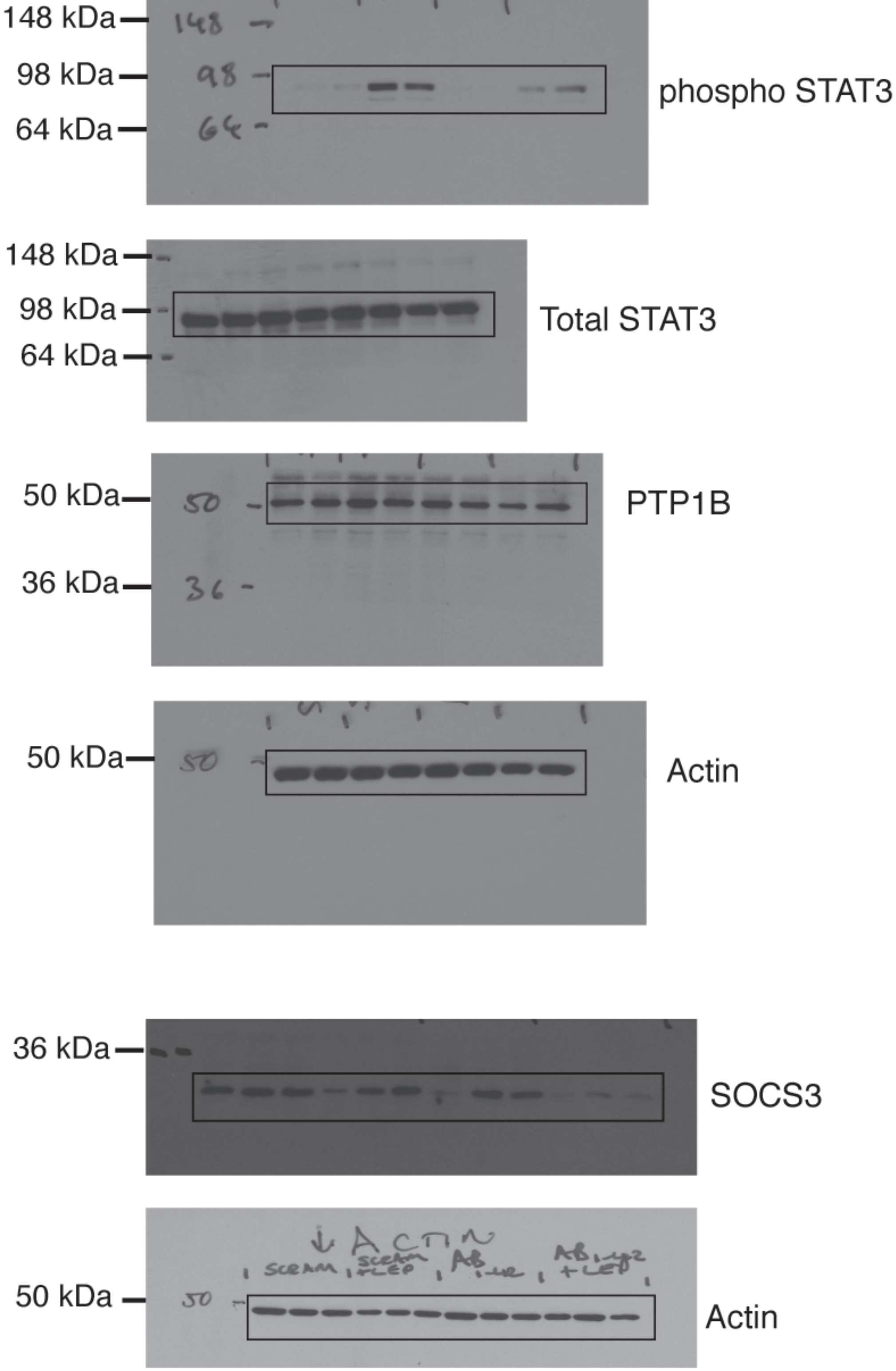

Supplementary Figure 11. Immunoblots that illustrate how icv A$\beta_{1\text{-}42}$ infusion alters p-STAT3, PTP1B and SOCS3 levels in response to leptin. These are uncropped immunoblot images corresponding to data shown in Figures 7f and 7i, respectively..

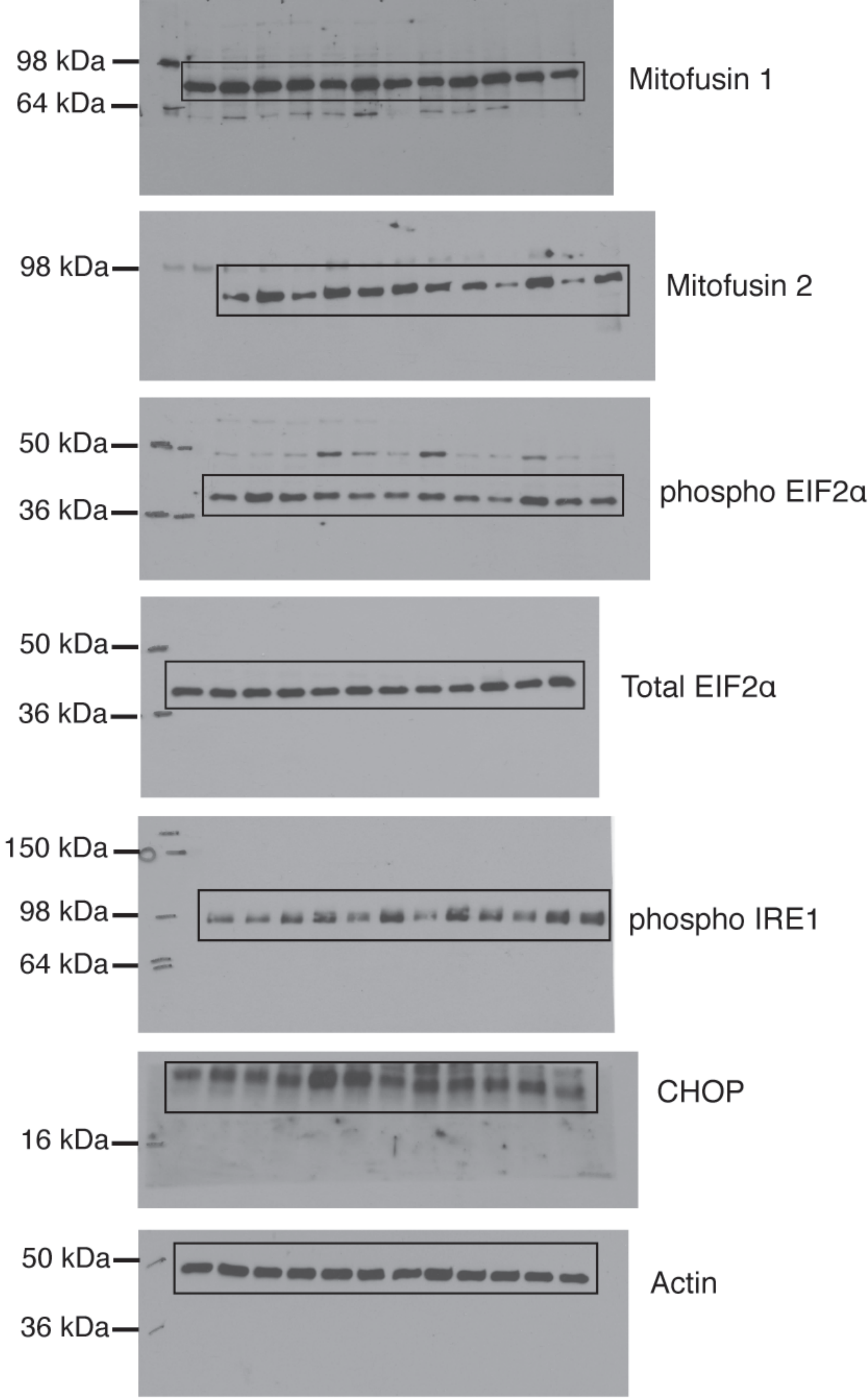

Supplementary Figure 12. Immunoblots that illustrate the lack of effect of loss of Bace1 on hypothalamic stress markers. These are uncropped immunoblot images corresponding to data shown in Supplementary Figure 3a.
